# Long-Term Effects of Early Life Seizures on Endogenous Local Network Activity of the Mouse Neocortex

**DOI:** 10.3389/fnsyn.2018.00043

**Published:** 2018-11-27

**Authors:** Pavlos Rigas, Charalambos Sigalas, Maria Nikita, Ani Kaplanian, Konstantinos Armaos, Leonidas Jordan Leontiadis, Christos Zlatanos, Aspasia Kapogiannatou, Charoula Peta, Anna Katri, Irini Skaliora

**Affiliations:** Biomedical Research Foundation of the Academy of Athens, Athens, Greece

**Keywords:** early-life, seizures, long-term effects, spontaneous, Up states, neocortex, mouse

## Abstract

Understanding the long term impact of early life seizures (ELS) is of vital importance both for researchers and clinicians. Most experimental studies of how seizures affect the developing brain have drawn their conclusions based on changes detected at the cellular or behavioral level, rather than on intermediate levels of analysis, such as the physiology of neuronal networks. Neurons work as part of networks and network dynamics integrate the function of molecules, cells and synapses in the emergent properties of brain circuits that reflect the balance of excitation and inhibition in the brain. Therefore, studying network dynamics could help bridge the cell-to-behavior gap in our understanding of the neurobiological effects of seizures. To this end we investigated the long-term effects of ELS on local network dynamics in mouse neocortex. By using the pentylenetetrazole (PTZ)-induced animal model of generalized seizures, single or multiple seizures were induced at two different developmental stages (P9–15 or P19–23) in order to examine how seizure severity and brain maturational status interact to affect the brain’s vulnerability to ELS. Cortical physiology was assessed by comparing spontaneous network activity (in the form of recurring Up states) in brain slices of adult (>5 mo) mice. In these experiments we examined two distinct cortical regions, the primary motor (M1) and somatosensory (S1) cortex in order to investigate regional differences in vulnerability to ELS. We find that the effects of ELSs vary depending on (i) the severity of the seizures (e.g., single intermittent ELS at P19–23 had no effect on Up state activity, but multiple seizures induced during the same period caused a significant change in the spectral content of spontaneous Up states), (ii) the cortical area examined, and (iii) the developmental stage at which the seizures are administered. These results reveal that even moderate experiences of ELS can have long lasting age- and region-specific effects in local cortical network dynamics.

## Introduction

The balance between excitatory and inhibitory synapses in the cortex is critical for normal brain function and adaptive behavior. In the developing brain this balance is shifted in favor of excitation due to the delayed maturation of inhibitory circuits (Gaiarsa et al., 1995; Ben-Ari, 2006); a fact that makes neonates and juveniles more susceptible to seizures (Hauser and Kurland, 1975; Olafsson et al., 2005), either spontaneous or in response to a number of different insults (Volpe, 1973). This becomes a clinical issue because early-life seizures are often associated with severe neurological and behavioral impairments in adult life, such as cognitive deficits and a higher propensity for epilepsy (Sillanpaa et al., 1998; Brunquell et al., 2002). However, the outcome of early-life seizures varies on an individual basis. Statistically, a third to half of affected children will fare well in adulthood, while the rest will either lead a sick life suffering from cognitive and neurological dysfunctions such as mental retardation, attention deficit disorders, behavioral disorders and epilepsy (17–40%) or will suffer premature death (16–30%) (Sillanpaa et al., 1998; Brunquell et al., 2002; Lombroso, 2007). This highly variable outcome emphasizes the need to understand the mechanisms that mediate the effects of seizures *per se*, i.e., dissociated from precursor and/or concurrent underlying pathologies, and from the effects of exposure to anti-epileptic drugs.

The study of the long-term effects of early-life seizures in humans is difficult and problematic due to the number of variables that influence the outcome (age of onset, etiology, seizure type, frequency and duration of seizures, genetics, environment, and pharmaceutical treatment), all of which are difficult to control in clinical studies (Haut et al., 2004; Holmes, 2005; Lombroso, 2007). Therefore, experimental models of early-life seizures are essential and rodents have been systematically used given the similarities to human seizures, in terms of electrical and behavioral parameters (Kubova and Moshe, 1994). For example, in both species, status epilepticus, the condition of persistent seizures, is manifested electrically with interictal and ictal discharges, and behaviorally with myoclonic seizures (Kubova and Moshe, 1994; Castro-Alamancos, 2000). In addition, humans and rodents have parallel behavioral profiles regarding the long-term effects of early seizures, as both develop cognitive deficits and a higher propensity for epilepsy (Holmes et al., 1998; Sillanpaa et al., 1998). Finally, just like humans, young rats and mice are much more prone to seizures than adults (Holmes, 2005).

Rodent studies have revealed a number of structural and/or functional effects of early-life seizures on the adult cortex, including: changes in neurogenesis (Holmes et al., 2002; Porter, 2008) or cell loss (Sankar et al., 2000) and synaptic re-organization (sprouting) of axons and terminals (Holmes and Ben-Ari, 1998; Holmes et al., 1998); modifications of glutamate and GABA receptors (Sanchez et al., 2001; Sogawa et al., 2001; Bo et al., 2004; Ni et al., 2004; Cornejo et al., 2007), changes in intrinsic properties (Villeneuve et al., 2000), or synaptic dynamics (Isaeva et al., 2006, 2009) of cortical cells, decreases in excitatory amino acid carrier (Zhang et al., 2004), and decreases in threshold for electrographic seizures (Santos et al., 2000; Isaeva et al., 2010). Moreover, respective behavioral studies in rodents have shown changes in behavior and cognition as reflected in deficits in learning and memory (Holmes et al., 1998; Huang et al., 1999; Chang et al., 2003; Karnam et al., 2009a,b) and sensory processing (Neill et al., 1996) indicating deficiencies in cortical function. However, the majority of these studies (a) have focused more on changes in either structure or behavior, rather than on alterations at the intermediate level of analysis, the physiology of neuronal circuits. This link is important in order to understand the underlying biological mechanisms that mediate early seizure effects. And (b) have focused much more on the effects of ELS on the hippocampus, rather than on the neocortex (Lombroso, 2007). Although this is understandable given the significance of the hippocampus in certain types of epilepsy, there is also evidence that brain regions differ, both in their sensitivity to seizures (Castro-Alamancos and Rigas, 2002; Rigas and Castro-Alamancos, 2004) and the resulting changes (Sankar et al., 1998; Kubova et al., 2001), implying that the effects of early-life seizures in the hippocampus cannot necessarily be generalized to the neocortex. This highlights an unmet need for studying the neocortex since this is the structure involved in most cognitive functions; in humans neonatal seizures typically involve the neocortex, and post-neonatal epilepsy is often of neocortical origin (Mizrahi and Clancy, 2000). Indeed, neonatal seizures more likely lead to epilepsy originating in neocortex than in hippocampus (Ronen et al., 2007). Therefore, progress in our understanding of the long-term effects of early seizures necessitates developing appropriate methods to evaluate the functional status of the neocortex (Lombroso, 2007).

Here we have examined the effect of chemically induced ELS on the endogenous cortical activity in brain slices from mouse cortex. Previous studies, including our own have shown that local recurrent networks formed by excitatory and inhibitory connectivity in the neocortex generate stable and self-sustaining periods of persistent activity alternating with periods of no activity, called *Up* and *Down* states, respectively – a prominent feature of the cortical activity during slow wave sleep *in vivo* (Steriade et al., 1993, 2001; Sanchez-Vives and McCormick, 2000; Cossart et al., 2003; MacLean et al., 2005; Haider et al., 2006; Rigas and Castro-Alamancos, 2007, 2009; Poulet and Petersen, 2008; Rigas et al., 2015; Sigalas et al., 2015, 2017). Such activity is maintained in cortical slice preparations, in the absence of sensory inputs or active neuromodulation, indicating that it is chiefly the outcome of intrinsic properties of local networks and hence reflects the ‘default’ activity of the cortex (Yuste et al., 2005; Sanchez-Vives et al., 2017). Since Up states are synaptically mediated network events that reflect the balance of excitation and inhibition in the neocortex (Sanchez-Vives and McCormick, 2000; Shu et al., 2003; Hasenstaub et al., 2005) we investigated whether and how they may reflect the long-term effects of ELS in an attempt to extend findings of an earlier report on the acute effects of seizures on this type of activity (Gerkin et al., 2010). Furthermore, in order to evaluate the differential spatial and temporal vulnerability to early life seizures, we examined two distinct cortical areas and induced the seizures at two different developmental stages.

## Materials and Methods

### Animals

C57Bl/6J mice were bred in the animal facility of the Center for Experimental Surgery of the Biomedical Research Foundation of the Academy of Athens. The facility is registered as a breeding and experimental facility according to the Presidential Decree of the Greek Democracy 160/91, which harmonizes the Greek national legislation with the European Council Directive 86/609/EEC on the protection of animals used for experimental and other scientific purposes. The present study was approved by the Regional Veterinary Service, in accordance to the National legal framework for the protection of animals used for scientific purposes (reference number 2834/08-05-2013). Mice were weaned at 27 days postnatally (P27, considering P0 as the day of birth), housed in groups of 5–10, in 267 mm × 483 mm × 203 mm cages supplied with bedding material and kept at a 12–12 dark-light schedule. Food was provided *ad libitum*.

### Seizure Induction

We induced generalized seizures in young mice by injecting intraperitoneally (i.p.) the proconvulsant pentylenetetrazole (PTZ), a GABA_A_R antagonist. This method has been widely used in chemically induced acute animal models of generalized seizures (Kubova and Moshe, 1994) and does not necessarily result in chronic epilepsy (Kandratavicius et al., 2014). PTZ can produce either non-convulsive absence seizures or myoclonic seizures and can even lead to status epilepticus (SE) if given at sufficient amounts (Loscher, 1997; Pitkaenen et al., 2006). Moreover, the protocol of PTZ delivery is simple and the compound can be easily administered intraperitoneally, subcutaneously or intravenously. A major advantage of using PTZ is the lack of neuron loss in developing rodents, even after the induction of recurrent seizures (Holmes et al., 1999). On the other hand, other chemical seizure models such as kainate (kainic acid) and pilocarpine produce wide-spread brain damage (Loscher, 1997). Kainic acid has a direct excitotoxic effect on neuronal cells that makes it difficult to separate it from the seizure-induced neuronal damage (Rao et al., 2006; Reddy and Kuruba, 2013), whereas pilocarpine has a pattern of neuronal damage similar to the kainic acid model with greater damage detected in the neocortex (Buckmaster et al., 2002; Reddy and Kuruba, 2013). Finally, PTZ has the advantage of being eliminated within 24 h from the animal without any known toxic or long-term direct effects (Loscher and Schmidt, 1988).

**FIGURE 1:**
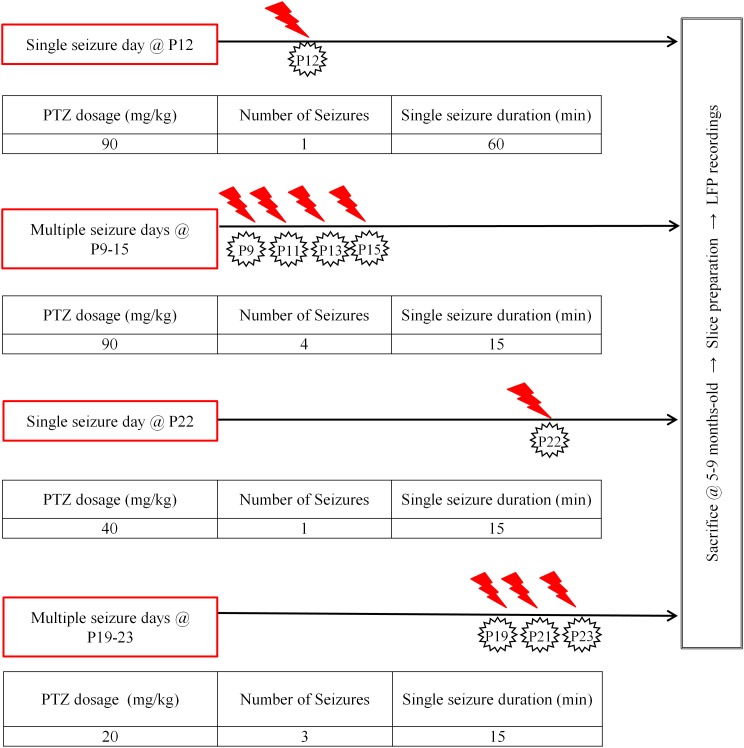
Seizure induction. Diagrammatic representation of our seizure induction scheme as applied at two distinct developmental ages (postnatal ages: 9–15 days old, P9–15 and 19–23 days old, P19–23), either once (single) or repetitively (multiple); at longer (60 min) or shorter (15 min) durations. Protocols were adjusted depending on age and times of injections in order to achieve highest survival rates for aimed seizure intensity and duration. Modified figure from Rigas et al. (2015) in Supplementary Figure 1.

Single or recurrent seizures were induced in mice during two defined developmental periods: P9–15 and P19–23, which are the mouse equivalent of neonatal period and early childhood in humans, respectively (Lombroso, 2007; Dutta and Sengupta, 2016). Control mice received an equal volume of saline (0.9%) delivered through the same number of injections as PTZ-treated mice (single or multiple, depending on the protocol). Although we did not perform any systematic electrophysiological assessment, PTZ treated mice appeared behaviorally indistinguishable from their saline-treated littermates, with no obvious signs of spontaneous seizures later in life, in line with previous reports (Kandratavicius et al., 2014). Mice responded to i.p. PTZ injections with a continuum of behaviors categorized into stages ranging from 1 to 6, which we briefly describe as follows in accordance with Luttjohann et al. (2009): Stage 1 was characterized by sudden behavioral arrest and/or motionless staring; stage 2 by facial jerking with muzzle or muzzle and eye; stage 3 by neck jerks; stage 4 by clonic seizure in a sitting position; stage 5 by convulsions including clonic and/or tonic–clonic seizures while lying on the belly and/or pure tonic seizures and stage 6 by convulsions including clonic and/or tonic–clonic seizures while lying on the side and/or wild jumping. Our injection scheme at both developmental stages involved either *single* or *multiple* seizures as described in Figure 1. We considered “seizure” the full-blown *generalized* tonic-clonic seizure for 15 min. In cases where this was not achieved with a single PTZ injection, additional injections were given in order for each mouse to experience the required duration of generalized (stage 5–6) seizures. In order to define the doses of PTZ which would induce seizures of stage 6 with the lowest mortality rate we performed pilot studies with mice at P12, P22 (single seizure tests) and P9–P15, P19–P23 (multiple seizure tests) which were injected intraperitoneally with various dosages of PTZ (*Standardization of Seizure Induction Protocol*). Repetitive seizures were induced every second day in order to increase survival rates. In line with previous work, we also found that P9–15 and P19–23 animals could not be given equivalent per kilogram doses (P9–15 vs. P19–23: 90 vs. 40 mg/kg, respectively) as the CD50 (Convulsion Dose 50%: dose producing clonic convulsions in 50% of tested animals) of PTZ increases to a peak at the animal age of 12 days and then declines to the CD50 of 8-days-old (Vernadakis and Woodbury, 1969; McCaughran and Manetto, 1982). Moreover, we found that, as opposed to younger ages, mice at P19–23 had to be injected with different doses of PTZ depending on whether we aimed at single or multiple seizures (single: 40 mg/Kg, multiple: 20 mg/Kg).

### Standardization of Seizure Induction Protocol

Pilot experiments were performed in order to optimize the seizure induction protocol in terms of efficiency and viability of seizure induction, a task which was often complicated by our equally important goals of increasing animal survival and reducing their suffering. For example, in our intial attempt to compare the effects of a single prolonged seizure (status epilepticus, SE) at P12 with those of multiple (five) SE occurring on five consecutive days from P10 to P15, we realized that when mice of the multiple protocol were left to recover on their own, survival rates were very low (3 out of 9 mice: 33.3%) already by the third day of sequential seizures. In addition, we observed that even animals that survived the seizures sometimes died after being returned to their cage, if they had not recovered completely. For this reason we decided to end seizures in all animals of this group by injecting them with the anticonvulsant diazepam (DZP, 2 mg/Kg) and aimed at five consecutive days of seizures. Despite this measure we ended up again with extremely low survival rates by the fifth day (2 out of 11 mice: 18.2%). In addition, we realized that PTZ was less effective in inducing stage 5–6 seizures when administered daily. In particular, as opposed to the first day of injections, stages 5–6 seizures were harder to achieve on subsequent days; in addition, seizures had delayed onsets and were of shorter durations. Therefore, we decided to administer PTZ every second day, each time aiming at seizures of a total duration of 15 min. With this method, PTZ-injections were very effective in inducing generalized seizures with hardly any need for supplementary injections. Moreover, in order to maintain the two ELS periods clearly separated, we decided to begin earlier (on P9 instead of P10). With these measures survival rates increased to 86.7% for up to the first four days of seizures. However, surprisingly, none of the mice could survive through a fifth day of seizures (on P17), a fact that forced us to limit our protocol between ages P9 and P15. A similar strategy was followed for seizure injections in mice of the older age group.

Although PTZ injections in our final protocols induced seizures in all mice, we observed a clear distinction between younger and older mice as well as between single and multiple days of PTZ-injections regarding (i) how easily seizures were induced and (ii) how long they lasted. In particular, P9–15 mice usually responded with a full-blown seizure to even a single PTZ injection and would remain in stage 5–6 for a protracted period of time (>1 h) during the single seizure protocol or the first day of a series of injections (multiple seizure protocol). In addition, although seizures in these mice during subsequent days of the multiple seizures protocol would last less than an hour, they still tended to remain in stage 5–6 significantly longer than 15 min. Therefore, we had to always inject these P9–15 PTZ-treated mice with DZP (2 mg/kg) in order to terminate seizures either at 60 min for status epilepticus (SE) or at 15 min for the shorter duration seizures protocols. In contrast, mice in the older group (P19–23) for both single and multiple seizures would almost always need supplementary doses in order to reach stage 5–6 seizures and/or sustain them for the aimed total seizure duration of 15 min. Although in older mice seizures lasted less and they showed earlier spontaneous recovery compared to younger mice, we still injected all of them with the anticonvulsant DZP (2 mg/kg) in order to terminate their seizures once they reached (i.e., either lasted or summed up to) 15 min. Finally, since saline-treated mice were not injected with DZP, in order to ensure that the electrophysiological phenotype of the PTZ-injected mice was not affected by DZP *per se*, we repeated the experiments in a separate group of animals that had received only DZP injections (2 mg/kg) and compared them to saline-treated mice. Importantly we found no differences in Up state activity between DZP- and saline-treated mice, suggesting that early-life administrations of DZP had no long-term effects on spontaneous cortical Up states (Supplementary Text, Section 1, Tables 1–8).

Younger and older mice also differed in lethality rates: P9–15 mice exhibited higher survival rates (90.9% for 1 × 60 min seizure at P12 and 86.7% for 4 × 15 min seizures at P9–15) compared to P19–23 mice (56.7% for 1 × 15 min seizure at P22 and 33.3% for 3 × 15 min seizures). In summary, protocols for seizure induction for the two age groups were adjusted accordingly in terms of both duration and number of seizures:

(a) The 60 min seizure was only applied in the younger mice. Instead, in P19–23 animals the single seizure duration was reduced to 15 min.

(b) Multiple seizures in both groups were induced on alternate days in order to increase survival rates. The number of seizures in the older group was restricted to three. As evident from the data (see Results section), this difference does not preclude valid comparisons between the two age groups.

(c) Given that four 15 min seizures in the P9–15 group had no effect on any of the parameters of Up state activity, we felt there was no justification to also include a group with one 15 min seizure in this age group.

### Brain Slice Preparation

**FIGURE 2:**
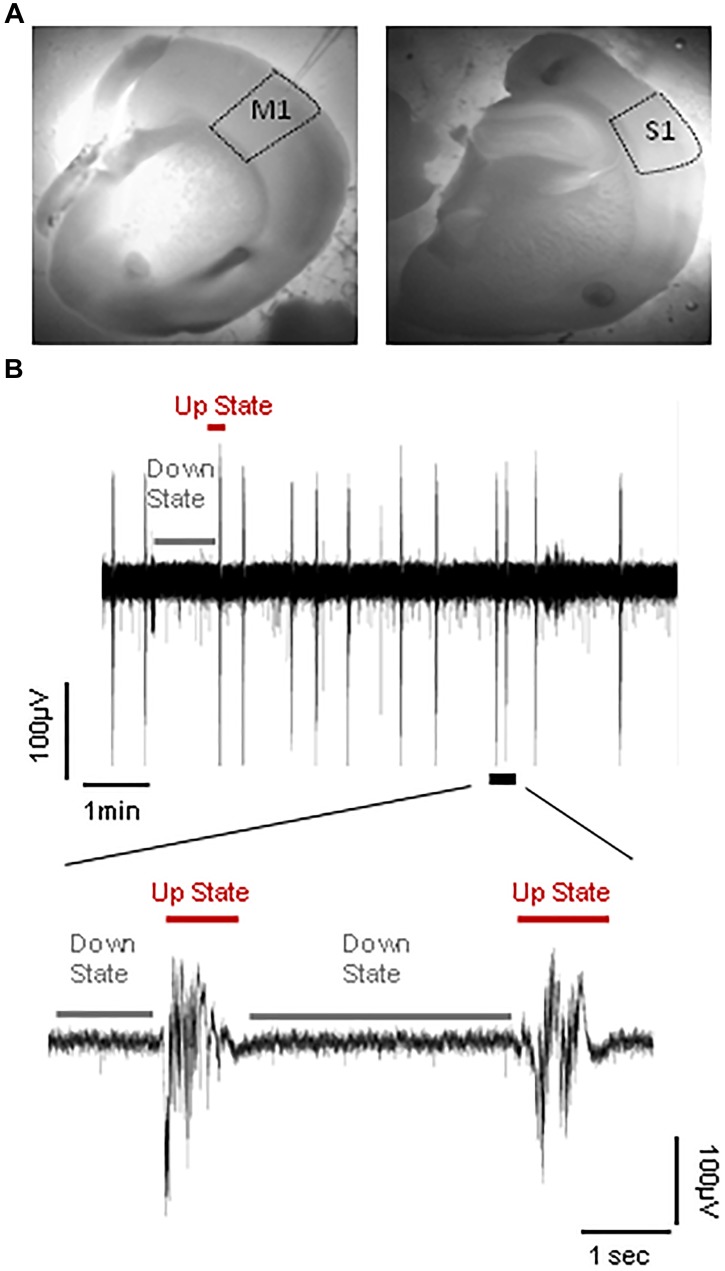
Spontaneous cortical Up states *in vitro*. **(A)** Outlined areas of primary motor (M1) and primary somatosensory area of the whiskers (S1) from which recordings were obtained. **(B)** *Upper trace*: typical example of continuous field potential recordings of alternating Up and Down states performed *in vitro* from the S1 cortical slice of an adult (5–9 months old) mouse. *Lower trace*: A close-up of two individual Up state events indicated in the trace above.

Coronal brain slices (400 μm) from primary somatosensory cortex of the whiskers [i.e., barrel cortex, S1BF; Anterior-Posterior from Bregma (A/P): 0.58–1.58 mm, Medial-Lateral (M/L): 2.5–4 mm] or primary motor cortex (M1; A/P: 1.54–0.74 mm, M/L: 1–2.75 mm) were prepared from the right hemisphere of adult male mice (5–9 months old) (Figure 2A). After the mouse was sacrificed with cervical dislocation, we removed the brain and placed it in an oxygenated (95% O_2_–5% CO_2_) ice-cold dissection buffer containing, in mM: KCl 2.14; NaH_2_PO_4_.H_2_O 1.47; NaHCO_3_ 27; MgSO_4_ 2.2; D-Glucose10; Sucrose 200; and CaCl_2_.2H_2_O 2; osmolarity (mean ± SD): 298 ± 5 mOsm, pH: 7.4. Brain slices were cut using a vibratome (VT 1000S, Leica) and placed in a holding chamber with artificial cerebrospinal fluid (ACSF) where they were left to recover at room temperature (RT: 24–26°C) for at least 1 h before transferred to the recording chamber. The ACSF contained (in mM): NaCl 126; KCl 3.53; NaH_2_PO_4_.H2O 1.25; NaHCO_3_ 26; MgSO_4_ 1; D-Glucose 10 and CaCl_2_.2H_2_O 2 [osmolarity (mean ± SD): 317 ± 4 mOsm, pH: 7.4].

### *In vitro* Electrophysiology

Following recovery, slices were transferred to a submerged type of chamber (Luigs and Neumann), where they were gravity-perfused at high flow rates (10–15 ml/min) to ensure optimal oxygenation of the cortical tissue (Hajos et al., 2009; Bregestovski and Bernard, 2012). Recordings were performed in “*in vivo* like” ACSF (whose composition was identical to above except for 1 instead of 2 mM CaCl_2_), since this ionic buffer is thought to better mimic cerebrospinal fluid *in vivo* (Fishman, 1992; Somjen, 2004) and we and others have previously shown that under these conditions cortical slices are spontaneously active in the form of a slow wave-like oscillation composed of alternating Up and Down states (Figure 2B; Sanchez-Vives and McCormick, 2000; MacLean et al., 2005; Rigas and Castro-Alamancos, 2007; Mann et al., 2009; Fanselow and Connors, 2010). Recordings were performed at RT after an hour (1 h) of incubation in 1 mM [CaCl_2_] ACSF buffer. To stabilize slices we modified our submerged chamber in order to included a surface of transparent silicone onto which up to four slices could be pinned. The advantage of this modification was that we could perform simultaneous recordings from different ages and/or brain regions and therefore maximize the yield of our experiments and achieve to directly compare different experimental groups under identical conditions.

Spontaneous network activity was assessed by means of local field potential (LFP) recordings (sampled at 10 kHz, band-passed filtered at 1 Hz–3 kHz) which were obtained from cortical layers II/III using low impedance (∼0.5 MΩ) glass pipettes filled with ACSF. Signals were acquired and amplified (MultiClamp 700B, Axon Instruments), digitized (InstruTech, ITC-18) and viewed on-line with appropriate software (AxoGraph). All reagents and drugs were purchased from Sigma except for KCl and K-gluconate, which were purchased from CARLO ERBA Reagents and Fluka, respectively.

### Data Analysis

For visualization and analysis of spontaneous LFP Up states, traces were exported to MATLAB format and analyzed with custom-made MATLAB scripts (LFPAnalyzer) that automatically detected the LFP events and marked their onsets and offsets as previously described (Rigas et al., 2015, 2017; Tsakanikas et al., 2017). In particular, preprocessing of the recordings included low-pass filtering at 200 Hz with a third order Butterworth filter and DC offset subtraction. Subsequently, detection of individual Up states was performed through the following automated steps: (a) the signal was transformed using the Hilbert Transform (Oppenheim and Schafer, 1998) and the Short-Time Energy Transform in parallel (Jalil et al., 2013), (b) a dynamic and data-driven threshold was then automatically estimated via Gaussian Mixture Modeling (McLachlan and Peel, 2000), and finally (c) the detected signal segments from each transformed signal were combined via an OR logical operation, resulting to the final LFP event (Tsakanikas et al., 2017).

In order to describe LFP Up states we employed a number of different parameters which were either measured or calculated and subsequently used for statistical analysis, as previously described (Rigas et al., 2015). In particular, home-made software was developed to automatically measure (i) *duration*, (ii) maximal negative peak (*amplitude*), (iii) *rectified area*, and (iv) *spectral power* of each detected Up state (Tsakanikas et al., 2017). Some of the measured parameters are depicted in Supplementary Figure 1. Furthermore, for each of our recordings we calculated (a) the *occurrence* of spontaneous events (i.e., number of events divided by the duration of the recording session) and (b) an overall Up state activity index calculated as the product of occurrence ^∗^ mean rectified area of Up states within each LFP recording (*Up state index*). Occurrence is a measure of how frequently spontaneous Up states occur while the rectified area is an overall measure of LFP Up state size, which includes both their duration and amplitude. Finally, the power spectrum of each event, estimated on the basis of Fourier Transform coefficients, is presented in the conventionally described frequency bands: *delta* (1–4 Hz), *theta* (4–8 Hz), *alpha* (8–12 Hz), *beta* (12–30 Hz), and *gamma* (30–100 Hz) range and normalized to the total power of each event in the 1–200 Hz range. The normalization procedure allows a direct comparison of the % differences of power, since LFP events within or between recordings can differ significantly in both amplitude and duration and thus in absolute power value.

### Statistical Analysis

Statistical analyses were performed using SPSS (version 17) software. Sample size was defined based on the number of slices and data were tested for normality using the Shapiro-Wilk test. Measurements of normally distributed data (*p* > 0.05) are presented as their mean ± standard deviation (SD), whereas data that deviated from normality are presented as their median and interquartile range. Factorial analysis of variance (ANOVA) for multiple group comparisons was applied to both normally and not normally distributed data after transforming data according to the rules of the Aligned Rank Transformation (Wobbrock et al., 2011) using the ARTool software^1^.

## Results

We investigated the long-term effects of early life seizures (ELS) on spontaneous cortical Up states during adulthood. ELS were introduced at two different developmental stages: either P9–15 or P19–23, and activity was sampled from two distinct cortical areas, namely the primary somatosensory cortex of the whiskers (barrel cortex, S1) and the primary motor cortex (M1) which differ in both their function and cytoarchitecture (Welker, 1971, 1976; Donoghue and Wise, 1982; Castro-Alamancos et al., 1995, 2007; Castro-Alamancos and Rigas, 2002; Katzel et al., 2011; Herculano-Houzel et al., 2013).

### The Long-Term Effects of Early-Life Status Epilepticus (SE) on Local Cortical Network Activity Are Region-Specific

**FIGURE 3:**
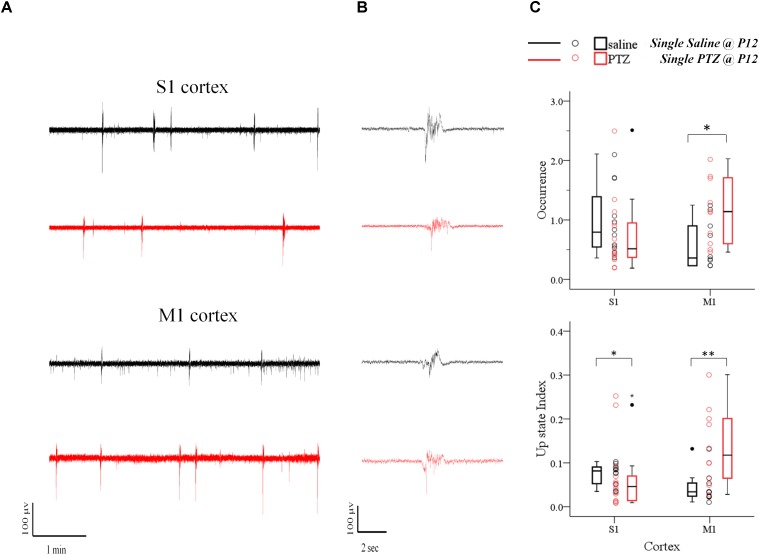
The long-term effects of status epilepticus on spontaneous Up states are region-specific. Prolonged (60 min) seizures (status epilepticus, SE) induced by a single administration of PTZ at P12 mice cause a region-specific effect in the adult cortex since they enhance spontaneous Up states in M1, but decrease network activity in S1 cortex compared to their control (saline-injected) groups. **(A)** Examples of continuous 5 min recordings of spontaneous Up states from S1 and M1 cortex of adult mice injected at P12 either with saline (black traces) or PTZ (red traces). **(B)** Close-up of individual Up states. The first Up state of respective long recording on the left was selected and demonstrated at higher time resolution. **(C)** Population data comparing occurrence (*upper panel*) and Up state index (an overall metric for spontaneous cortical activity that integrates the occurrence with the rectified area of Up state events, *lower panel*) of spontaneous Up states in adult mice which had experienced SE at P12 with age-matched control mice. *Open circles* represent superimposed vertical scatterplots of individual data points of the control (saline, *black*) and treated (PTZ, *red*) groups of animals. *Box and Whisker plots* placed on each side of the scatterplots describe the spread of data for the saline- (black) and PTZ-treated (red) group showing interquartile ranges (length of *boxes*), upper and lower limits of data (*whiskers*), median (*line inside box*), extreme values (*stars*), and outliers (*filled circles*). *Asterisks* indicate levels of statistically significant differences between compared groups (^∗^*p* < 0.05, ^∗∗^*p* < 0.01).

We first examined the effect of a 60 min status epilepticus (SE). SE has been defined as a state of continuous or recurrent seizures for at least 30 min with incomplete or no recovery between seizures (Lowenstein et al., 1999; Mitchell, 2002; Gaitanis and Drislane, 2003; Varelas and Mirski, 2009). This experiment was only possible during the earlier postnatal period (P9–15) because at later stages (P19-23) animal lethality was prohibitive. A two-way analysis of variance (two-way ANOVA) was conducted on the influence of two independent variables (seizures and cortex) on network dynamics of local spontaneous Up states as described by ten parameters, namely: occurrence, duration, amplitude, rectified area, Up state index, normalized delta, normalized theta, normalized alpha, normalized beta and normalized gamma (as described in *Materials and Methods*). *Seizures* included two levels [no seizures (“injections with saline”) and seizures (“injections with PTZ”)] and *cortex* also consisted of two levels (S1, M1). There was a highly significant interaction between the effects of seizure and cortex for occurrence [*F*(1,42) = 9.071, *p* = 0.004] and Up states index of spontaneous activity [*F*(1,42) = 12.7, *p* = 0.001]; and a marginally significant effect of the two on Up state amplitude [*F*(1,42) = 4.172, *p* = 0.047] and rectified area [*F*(1,42) = 4.151, *p* = 0.048] (Supplementary Text, Section 2.1.2, Table 9). Further simple effect analyses showed that SE during early life significantly increased the occurrence of spontaneous Up states in the adult M1 but not S1 cortex [Occurrence: *S1, saline (N*_Animals_ *= 8, N*_slices_ *= 12) vs. PTZ (N*_Animals_ *= 9, N*_slices_ *= 14): 0.80 (1.01) vs. 0.512 (0.64) Up states/min [median (interquartile range)], F(1,42) = 2.502, p = 0.121, Bonferroni; M1, saline (N*_Animals_ *= 9, N*_slices_ *= 10) vs. PTZ (N*_Animals_ *= 7, N*_slices_ *= 10): 0.36 (0.74) vs. 1.14 (1.14) Up states/min [median (interquartile range)], F(1,42) = 4.425, p = 0.041, Bonferroni*, Figure 3 and Supplementary Text, Section 2.2.1.1]. The same analysis applied to Up state index (which reflects an overall metric for spontaneous cortical activity that integrates the occurrence with the rectified area of Up state events) confirmed the enhancement Up states dynamics in M1 but also revealed a significant effect in the opposite direction for S1 [Up state index: *S1, saline vs. PTZ: 0.08 (0.04) vs. 0.05 (0.06) [median (interquartile range)], F(1,42) = 5.576, p = 0.023, Bonferroni; M1, saline: 0.03 (0.03) vs. 0.12 (0.15) [median (interquartile range)], F(1,42) = 7.601, p = 0.009, Bonferroni*, Figure 3 and Supplementary Text, Section 2.2.1.2]. On the contrary there was no significant interaction between the effects of seizures and cortex type for duration or spectral content of spontaneous Up states (Supplementary Text, Section 2.1.2., Table 9) and early life SE had no main effect on either Up state duration or spectral power (Supplementary Text, Section 2.3., Table 10). Taken together, these results indicate a region-specific long-term effect of prolonged status epilepticus in early life on the adult cortex.

### The Long-Term Effects of ELS on Local Cortical Network Activity Depend on the Duration of Individual Seizures, Rather Than on Their Frequency

**FIGURE 4:**
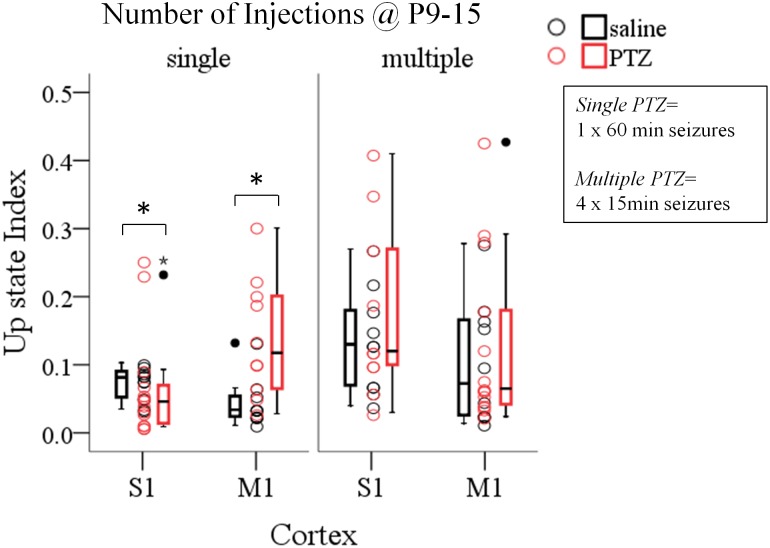
The long-term effects of early life seizures on spontaneous Up states depend on their pattern. Single 60 min seizures, but not multiple seizures of equal total duration (4 min × 15 min) during early life (P9–15) significantly affect spontaneous Up states in the adult cortex. *Open circles* represent superimposed vertical scatterplots of individual data points of the control (saline, *black*) and treated (PTZ, *red*) groups of animals. *Box and Whisker plots* placed on each side of the scatterplots describe the spread of data for the saline- (black) and PTZ-treated (red) group showing interquartile ranges (length of *boxes*), upper and lower limits of data (*whiskers*), median (*line inside box*), extreme values (*stars*), and outliers (*filled circles)*. *Asterisks* indicate levels of statistically significant differences between compared groups (^∗^*p* < 0.05).

Having documented the significant impact of prolonged ELS (60 min status epilepticus, SE) on the adult cortex we then wondered if this effect was dependent on the pattern and/or total duration of SE, i.e., whether multiple shorter duration seizures would also affect cortical network dynamics as single long-lasting SE did. This was prompted by the fact that, in humans, the outcome of febrile seizures, commonly triggered by fever in infants and children, critically depends on their duration – with short seizures being benign compared to prolonged seizures (Annegers et al., 1987; French et al., 1993; Verity et al., 1998). Therefore, in a second group of pups we induced four 15 min seizures on every second day from P9–P15 and we tested their effect on spontaneous Up states during adulthood. To this end we conducted a three-way ANOVA on the influence of three independent variables (*injections, number of injections* and *cortex*) on the dynamics of spontaneous Up states. As previously described *injections* included two levels [“injections with saline” (i.e., no seizures) and “injections with PTZ” (i.e., seizures)], *cortex* consisted of two levels (S1, M1) and *number of injections* also had two levels (single and multiple). We found a significant interaction among the three factors for the overall index of spontaneous Up states (*Up state index*) [*F*(1,82) = 4.458, *p* = 0.038], but not for the other parameters tested (Supplementary Text, Section 3.1, Tables 11–13). Simple effect analysis revealed a significant effect of a single prolonged seizure on Up state network index in both cortices, as opposed to multiple seizures of equal total duration which had no effect on either cortex [Single Seizures: *S1, saline (N*_Animals_ *= 8, N*_slices_ *= 12) vs. PTZ (N*_Animals_ *= 9, N*_slices_ *= 14): 0.08 (0.04) vs. 0.05 (0.06) [median (interquartile range)], F(1,82) = 5.154, Bonferroni, p = 0.026; M1, saline (N*_Animals_ *= 9, N*_slices_ *= 10) vs. PTZ (N*_Animals_ *= 7, N*_slices_ *= 10): 0.03 (0.03) vs. 0.12 (0.15) [median (interquartile range)], F(1,82) = 7.025, Bonferroni, p = 0.010*, Figure 4; Multiple Seizures: *S1, saline (N*_Animals_ *= 7, N*_slices_ *= 10) vs. PTZ (N*_Animals_ *= 7, N*_slices_ *= 10): 0.13 (0.12) vs. 0.12 (0.20) [median (interquartile range)], F(1,82) = 0.122, Bonferroni, p = 0.728; M1, saline (N*_Animals_ *= 6, N*_slices_ *= 10) vs. PTZ (N*_Animals_ *= 9, N*_slices_ *= 14): 0.07 (0.14) vs. 0.07 (0.16) [median (interquartile range)], F(1,82) = 0.600, Bonferroni, p = 0.441*, Figure 4 and Supplementary Text, Section 3.2.1.1]. Finally, a two-way ANOVA of the effects of *number of injections* and *injections* revealed no significant interaction for any Up states variable that we tested (Supplementary Text, Section 3.3, Tables 14–16). Therefore, although both protocols of induced seizures that we applied (single and multiple) were of equal total duration (4 min × 15 min vs. 60 min) our results indicate that long-lasting single seizures rather than multiple shorter seizures cause long-term effects in network dynamics emphasizing the importance of ELS duration over ELS frequency in determining their long-term impact.

### Younger Ages Are More Resilient to the Long-Term Effects of ELS on Local Cortical Network Activity

**FIGURE 5:**
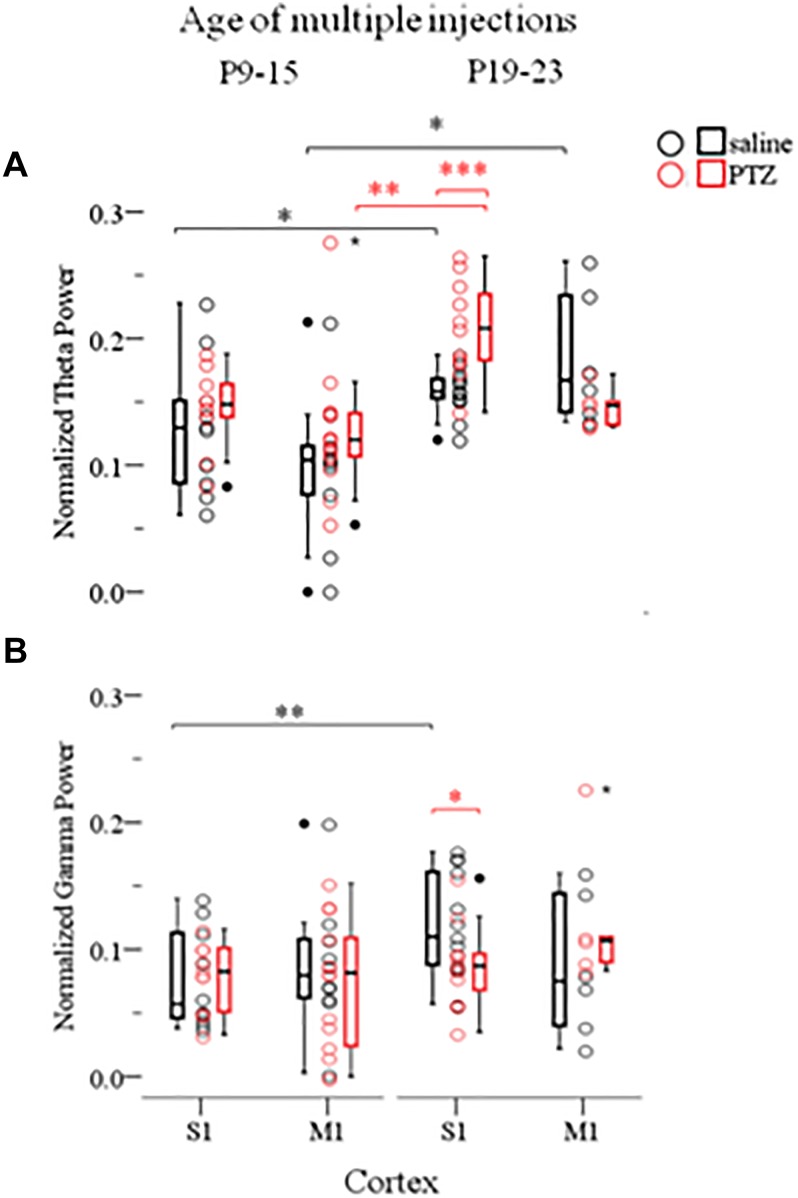
The long-term effects of multiple ELSs on spontaneous Up states are age-specific. Multiple 15 min PTZ-induced seizures administered at P9–15 mice had no effect on the spectral content of spontaneous Up states in the theta **(A)** (4–8 Hz) and gamma **(B)** (30–100 Hz) range, in either primary sensory (S1) or motor (M1) cortex. On the contrary, multiple seizures at P19–25 significantly increased theta power, but reduced gamma power in S1, but not M1, cortex. Spectral power of Up states within each range was normalized to the total Up state power in the 1–200 Hz range. *Open circles* represent superimposed vertical scatterplots of individual data points of the control (saline, *black*) and treated (PTZ, *red*) groups of animals. *Box and Whisker plots* placed on each side of the scatterplots describe the spread of data for the saline- (black) and PTZ-treated (red) group showing interquartile ranges (length of *boxes*), upper and lower limits of data (*whiskers*), median (*line inside box*), extreme values (*stars*), and outliers (*filled circles*). *Asterisks* indicate levels of statistically significant differences between compared groups (^∗^*p* < 0.05, ^∗∗^*p* < 0.01, ^∗∗∗^*p* < 0.001).

In order to investigate whether the effects of ELS on cortical network dynamics are age dependent, and given that the 60 min SE was not possible in older animals due to increased mortality rates (as described in *Materials and Methods*), we compared the effects of multiple seizures of shorter duration, implemented at either P9–15 or P19–23, in both cortices. For this we conducted a three-way ANOVA as before, of the effect of three independent variables: seizures (*injections*: saline and PTZ), age of seizures (*age of injections*: P9–15 and P19–23) and type of cortex (*cortex*: S1 and M1) on Up states dynamics as quantified by respective parameters. We found a significant interaction of the effects of these three factors on Up state spectral content at the theta [*F*(1,71) = 4.620, *p* = 0.035] and gamma [*F*(1,71) = 4.184, *p* = 0.045] range (Supplementary Text, Section 4.1, Tables 17–19). Subsequent simple effect analysis of *seizures* on the theta power revealed a significant age-specific increase of power after seizures induced at P19–23 but not at P9–15. In addition, this effect was region-specific since it occurred in S1 but not M1 cortex [P19-23: *S1, saline (N*_Animals_ *= 7, N*_slices_ *= 13) vs. PTZ (N*_Animals_ *= 6, N*_slices_ *= 11):* 0.16 (0.02) *vs.* 0.21 (0.06) *[median (interquartile range)], F(1,71) = 14.206, Bonferroni, p < 0.001; M1, saline (N*_Animals_ *= 5, N*_slices_ *= 6) vs. PTZ (N*_Animals_ *= 4, N*_slices_ *= 5):* 0.17 (0.10) *vs.* 0.15 (0.03) [*median (interquartile range)], F(1,71) = 0.458, Bonferroni, p = 0.501*, Figure 5; P9–15: *S1, saline (N*_Animals_ *= 7, N*_slices_ *= 10) vs. PTZ (N*_Animals_ *= 7, N*_slices_ *= 10):* 0.13 (0.08) *vs.* 0.15 (0.04) *[median (interquartile range)], F(1,71) = 1.244, Bonferroni, p = 0.269; M1, saline (N*_Animals_ *= 6, N*_slices_ *= 10) vs. PTZ (N*_Animals_ *= 9, N*_slices_ *= 14):* 0.10 (0.06) *vs.* 0.12 (0.04) *[median (interquartile range)], F(1,71) = 2.682, Bonferroni, p = 0.106*, Figure 5 and Supplementary Text, Section 4.2.1.1]. A similar age- and region-specific effect of early multiple seizures on the adult cortex was also supported by respective simple effect analysis of seizures on the gamma power of Up states. In particular we found that multiple ELS significantly reduced the power of gamma frequencies in the adult S1, but not M1 cortex, when they occurred at later (P19–23) rather than earlier (P9–15) developmental stages [P19–23: *S1, saline (N*_Animals_ *= 7, N*_slices_ *= 13) vs. PTZ (N*_Animals_ *= 6, N*_slices_ *= 11):* 0.11 (0.08) *vs.* 0.09 (0.04) *[median (interquartile range)], F(1,71) = 4.750, Bonferroni, p = 0.033; M1, saline (N*_Animals_ *= 5, N*_slices_ *= 6) vs. PTZ (N*_Animals_ *= 4, N*_slices_ *= 5):* 0.08 (0.11) *vs.* 0.11 (0.08) [*median (interquartile range)], F(1,71) = 0.391, Bonferroni, p = 0.534*, Figure 5; P9–15: *S1, saline (N*_Animals_ *= 7, N*_slices_ *= 10) vs. PTZ (N*_Animals_ *= 7, N*_slices_ *= 10):* 0.06 (0.07) *vs.* 0.08 (0.05) *[median (interquartile range)], F(1,71) = 0.081, Bonferroni, p = 0.777; M1, saline (N*_Animals_ *= 6, N*_slices_ *= 10) vs. PTZ (N*_Animals_ *= 9, N*_slices_ *= 14):* 0.08 (0.05) *vs.* 0.08 (0.09) [*median (interquartile range)], F(1,71) = 0.391, Bonferroni, p = 0.534*, Figure 5 Supplementary Text, Section 4.2.1.2].

A simple effect analysis of the *age of injections* revealed a significant effect of age within the saline- and PTZ-treated groups for both theta and gamma power. In particular we found theta power of spontaneous Up states to be significantly higher in both S1 and M1 cortex of the adult mice that were injected with saline at P19-23 compared to saline-treated mice at P9–15 [Theta*: S1, saline, P9–15 (N*_Animals_ *= 7, N*_slices_ *= 10) vs. P19–23 (N*_Animals_ *= 7, N*_slices_ *= 13): 0.13 (0.08) vs. 0.16 (0.02) [median (interquartile range)], F(1,71) = 6.175, Bonferroni, p = 0.015; M1, saline, P9–15 (N*_Animals_ *= 7, N*_slices_ *= 10) vs. P19–23 (N*_Animals_ *= 7, N*_slices_ = 13): 0.10 (0.06) vs. 0.17 (0.10) [median (interquartile range)], F(1,71) = 6.006, Bonferroni, p = 0.017, Figure 5 and Supplementary Text, Section 4.2.2.1]. Respectively, we found that gamma power of spontaneous Up states in the adult S1 cortex was significantly lower in mice injected with saline at P19-23 compared to mice injected with saline at P9–15 [Gamma: *S1, saline, P9–15 (N*_Animals_ *= 7, N*_slices_ *= 10) vs. P19–23 (N*_Animals_ *= 7, N*_slices_ *= 13):* 0.06 (0.07) *vs.* 0.11 (0.08) *[median (interquartile range)], F(1,71) = 7.589, Bonferroni, p = 0.007*, Figure 5 and Supplementary Text, Section 4.2.2.2]. This age-dependent saline effect was an unexpected finding for which we have no satisfactory explanation given that recording conditions in all animal groups were identical. Moreover, this result compromises the aforementioned age-specific effect of early seizures on the gamma power of Up states in the adult S1 cortex and renders it inconclusive since simple effect analysis of age showed that gamma levels in adult mice treated with PTZ at P19-23 did not differ significantly from those that had received PTZ at P9–15 [Gamma: *S1, PTZ, P9–15 (N*_Animals_ *= 7, N*_slices_ *= 10) vs. P19–23 (N*_Animals_ *= 7, N*_slices_ *= 13):* 0.08 (0.05) *vs.* 0.09 (0.04) *[median (interquartile range)], F(1,71) = 0.373, Bonferroni, p = 0.543*, Figure 5 and Supplementary Text, Section 4.2.2.2]. On the contrary, we would argue that the age- (and cortex-) specific effect of ELS on the theta content of spontaneous Up states in the adult S1 cortex, as concluded from our three-way ANOVA analysis, is a reliable result despite the age-dependent saline-effect revealed by simple effect analysis, since PTZ injections at P19–23 increased theta power in the adult S1 (but not M1) cortex compared not only to saline treatment at the same age, but also to PTZ injections at P9–15 [Theta: S1, PTZ, P9–15 *(N*_Animals_ *= 7, N*_slices_ *= 10) vs. P19–23 (N*_Animals_ *= 7, N*_slices_ *= 13):* 0.15 (0.04) *vs.* 0.21 (0.06) *[median (interquartile range)], F(1,71) = 12.938, Bonferroni, p = 0.001; M1, PTZ*, P9–15 *(N*_Animals_ *= 7, N*_slices_ *= 10) vs. P19–23 (N*_Animals_ *= 7, N*_slices_ *= 13):* 0.12 (0.04) *vs.* 0.15 (0.03) *[median (interquartile range)], F(1,71) = 3.872, Bonferroni, p = 0.053*, Figure 5 and Supplementary Text, Section 4.2.2.1]. Finally, a two-way ANOVA of the effects of *age of injections* and *injections* on the remaining Up states variables (i.e., except for theta) revealed no significant interaction (Supplementary Text, Section 4.3, Tables 20–22). In conclusion, these results indicate older ages to be more vulnerable to the long-term effects of ELS, while they provide additional evidence that the effects of ELS are region specific.

### The Long-Term Effects of ELS on Cortical Dynamics Depend on Their Frequency

**FIGURE 6:**
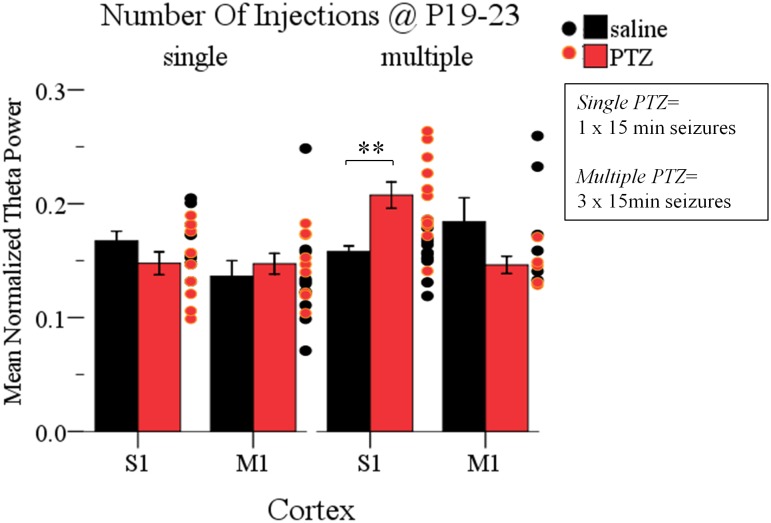
The long-term effects of early life seizures on spontaneous Up states depend on their frequency. Multiple, but not single, 15 min seizures administered in P19-23 mice have a region-specific effect on the adult cortex since they significantly increased the theta (4–8 Hz) content of spontaneous Up states in the adult S1, but not M1, cortex compared to saline-treated groups. Spectral power of Up states in the theta range was normalized to the total Up state power in the 1–200 Hz range. *Dots* represent superimposed vertical scatterplots of individual data points of the control (saline, *black*) and treated (PTZ, *red*) groups of animals compared on the left. *Asterisks* indicate levels of statistically significant differences between compared groups (^∗∗^*p* < 0.01).

Finally, having documented that multiple intermittent seizures at P19-23 significantly enhance Up state theta power in the adult cortex, we asked whether a single seizure of the same duration (15 min) was enough to produce a similar effect. To this end we conducted a three-way ANOVA of the effect of the three independent variables: seizures (*injections*: saline and PTZ), number of seizures (*number of injections*: single and multiple) and type of cortex (*cortex*: S1 and M1) on Up states dynamics. We found a significant interaction of the effects of these three factors, which was specific for the spectral content of spontaneous Up states at the theta range [*F*(1,66) = 13.195, *p* = 0.001] (Supplementary Text Section 5.1, Tables 23–25). Subsequent simple effect analysis of *seizures* (*injections*) revealed a frequency-specific effect of seizures since multiple but not single seizures significantly increased the theta power in the adult cortex. In addition, this effect was cortex-specific since it was significant for S1 but marginally non-significant for M1 [Single: *S1, saline (N*_Animals_ *= 7, N*_slices_ *= 9) vs. PTZ (N*_Animals_ *= 8, N*_slices_ *= 10):* 0.17 ± 0.03 *vs.* 0.15 ± 0.03 *(mean ± SD), F(1,66) = 1.637, Bonferroni, p = 0.205; M1, saline (N*_Animals_ *= 8, N*_slices_ *= 11) vs. PTZ (N*_Animals_ *= 7, N*_slices_ *= 9):* 0.14 ± 0.05 *vs.* 0.15 ± 0.03 *(mean ± SD), F(1,66) = 0.523, Bonferroni, p = 0.472;* Multiple: *S1, saline (N*_Animals_ *= 7, N*_slices_ *= 13) vs. PTZ (N*_Animals_ *= 6, N*_slices_ *= 11):* 0.16 ± 0.02 *vs.* 0.21 ± 0.04 *(mean ± SD), F(1,66) = 13.157, Bonferroni, p = 0.001; M1, saline (N*_Animals_ *= 5, N*_slices_ *= 6) vs. PTZ (N*_Animals_ *= 4, N*_slices_ *= 5):* 0.18 ± 0.05 *vs.* 0.15 ± 0.02 *(mean ± SD), F(1,66) = 3.525, Bonferroni, p = 0.065;* Figure 6 and Supplementary Text, Section 5.2.1.1]. Finally, in order to test for potential cortical-independent effects of severity of seizures (number of injections) on Up states, we conducted a two-way ANOVA of the effects of *number of injections* and *injections* on the remaining Up states variables (i.e., except for theta), which, however, revealed no significant interaction (Supplementary Text, Section 5.3, Tables 26–28). Overall our results show that the impact of short duration ELS increases with their frequency.

Taken together, our results provide evidence that early-life seizures can affect spontaneous local network activity recorded even months later, in the adult mouse neocortex. Importantly, we found this effect to vary depending on several factors such as: (a) the severity of seizures, (b) the cortical area tested, (c) the age at which seizures occurred, and finally (d) the physiological parameter measured (Table 1). We conclude that long-term effects of ELS on cortical network dynamics are *region-specific* and *age-specific*. In particular, prolonged single status epilepticus -but not multiple intermittent seizures- during the second postnatal week (P12) enhanced spontaneous Up states in motor but reduced those in sensory cortex. However, multiple intermittent seizures during the third (P19–23) but not the second (P9–15) postnatal week affected S1, but not M1, cortical networks by increasing their theta activity.

**Table 1:** Summary of the effect of ELS at two developmental stages (P9–15 and P19–23) on spontaneous Up states in M1 and S1 cortex of the adult mouse.

		Age of seizures induction			

		P9–15		P19–23	
			
Cortex		S1	M1	S1	M1
Type of seizure	Single 60 min	↓Up state index	↑Occurrence ↑Up state index		
	Single 15 min			*No effect*	*No effect*
	Multiple 15 min	*No effect*	*No effect*	↑Theta	*No effect*


*Symbols indicate whether there was an increase (↑), decrease (↓) on respective parameter.*

## Discussion

### Endogenous Cortical Network Activity in the Form of Spontaneous Up States Is a Sensitive Neurophysiological Measure to Reflect Long-Term Effects of Early Life Seizures on the Cerebral Cortex

In this study we have shown that ELS can cause permanent changes in cortical network dynamics persisting well into adulthood. Experimentally induced seizures are routinely used to explore the impact of ELS on the developing brain. However, the results of such studies are often contradictory and have failed to provide a broad consensus on the effects of ELS on brain function and structure, or behavior (Wasterlain, 1997; Lado et al., 2000). This is at least partly due to the wide variability in experimental protocols including (a) the specific ages at which seizures are induced; (b) the seizure-induction methods (e.g., chemically, electrically, febrile-, and hypoxia-induced seizures); (c) the number of seizures (single vs. multiple); (d) the duration of individual seizures [prolonged (i.e., status epilepticus) vs. intermittent]; (e) the post-seizure intervals at which effects are investigated (immediate, medium- or long-term); and (f) the brain area under investigation. Furthermore, most studies that examine the effects of ELS focus on specific neuronal and/or synaptic elements, or overall behavioral phenotypes, rather than on the intermediate level of organization such as the neuronal network. Here, using the PTZ model we investigated how experimental seizures of distinct duration, number and age at which they occurred affected two distinct cortical areas during adulthood. To our knowledge this is the first attempt to examine the long-term outcome of ELS as a result of the independent or interactive effects of distinct factors, such as seizure severity, brain region and age of seizure onset, all tested within the same study.

It is also the first study to quantify the *long-term* effects of ELS on the endogenous cortical network activity in the form of recurring Up/Down states. This type of activity is characteristic of the slow oscillation (SO) and is observed both *in vivo*, during quiescence, but also *in vitro*, in the brain slice preparation (Steriade et al., 1993, 2001; Sanchez-Vives and McCormick, 2000; Cossart et al., 2003; MacLean et al., 2005; Haider et al., 2006; Rigas and Castro-Alamancos, 2007, 2009; Poulet and Petersen, 2008). The slow oscillation is considered the ‘default’ activity of the cortex rendering it a *central pattern generator* (Yuste et al., 2005; Sanchez-Vives et al., 2017). Hence, the SO and in particular its active components (i.e., the spontaneous Up states) provide the framework to assess the impact of early life events on local network dynamics of the adult cerebral cortex. Since Up states consist an emergent property of cortical microcircuits that involve both excitatory and inhibitory neurons (Steriade et al., 1993) they reflect the balanced contribution of excitation and inhibition of cortical networks (Sanchez-Vives and McCormick, 2000; Shu et al., 2003; Hasenstaub et al., 2005; Haider et al., 2006). To our knowledge this is the first report that ELS can have a long-term impact on spontaneous Up states, extending findings of an earlier report on the acute effects of seizures on this type of activity (Gerkin et al., 2010).

### The Effects of ELS Are Regionally Specific

Our results suggest that ELS affect the adult brain in a region-specific manner. In particular, we found a significant effect of cortex in both our main results: (a) prolonged seizures (status epilepticus, SE) at P12 significantly affected both primary motor (M1) and sensory (S1) cortices, but in opposite directions since they enhanced the occurrence and overall index of spontaneous Up states in M1, but decreased Up states index in S1. And (b) multiple intermittent seizures at P19-23 affected S1 but not M1 cortex in terms of the theta content of their Up states.

The idea that ELS may affect the brain in a region-specific manner is supported by a recent study showing that seizure activity in the early postnatal mouse neocortex for 2 h diminishes the rates of apoptosis in M1 but not S1 (Blanquie et al., 2017). Apoptosis plays a critical role in establishing neural circuits in the developing mammalian brain, by selectively eliminating a substantial portion of the initially overproduced neurons through cell death (Haydar et al., 1999). Moreover, there is a general consensus that spontaneous electrical activity, a widespread property of the developing cerebral cortex, plays a pro-survival role, while reduced activity increases the number of neurons undergoing apoptosis (Ikonomidou et al., 1999; Golbs et al., 2011; Murase et al., 2011; Lebedeva et al., 2017). Therefore, in regard with our own results, prolonged seizures (status epilepticus, SE) at P12 could for example compromise apoptotic processes in M1 specifically, leading to larger and/or denser neuronal assemblies in the cortex of PTZ-treated compared to non-treated mice, capable of producing spontaneous network events (i.e., Up states) at higher incidences. Moreover, evidence suggests that, as opposed to primary pyramidal neurons, the cell fate of developing cortical inhibitory interneurons is rather intrinsically programmed than activity-dependent (Sahara et al., 2012; Southwell et al., 2012). This property of inhibitory cells could, therefore, render them resistant to the excessive neural activity of seizures, thus, preserving them to normal adult levels. Such a scenario would explain to an extent why we found early SE to affect the occurrence of Up states but not their duration, since inhibition has been associated with modulation of Up state duration (Mann et al., 2009; Sanchez-Vives et al., 2010). Similar to our results, which differentiate the effect of early seizures depending on the variable of network dynamics tested, 20–40 min long seizures in 2 week old mice increased the occurrence of spontaneous Up states in the somatosensory cortex 24 h later without affecting their duration (Gerkin et al., 2010). The differential effect of ELS on Up state occurrence and duration could reflect the fact that previous studies, including our own, have shown that the mechanism of Up state generation (which is reflected in the Up state occurrence) is distinct from the mechanism of Up state termination (which is reflected in the Up state duration) (Mann et al., 2009; Sanchez-Vives et al., 2010; Sigalas et al., 2015). Finally, enhanced Up state activity in M1 cortex suggests an increased excitability of this cortical area which at its extreme could serve as the substrate for the development of spontaneous seizures (Ziburkus et al., 2013). Although none of our PTZ-treated mice appeared to develop epileptic behavior, ELS have been associated with epileptogenesis during adulthood (Sillanpaa et al., 1998; Brunquell et al., 2002). On the other hand, the reduced index of spontaneous Up states (*Up state index*) in S1 recorded in the brain of adult mice that had experienced SE indicates compromised network dynamics which, in turn, could contribute to sensory processing and cognitive deficits associated with ELS (Neill et al., 1996; Holmes, 2016).

Our findings on the region-specific effects of ELS on the cerebral cortex suggest that the general concept of the “immature brain” may not be sufficient to predict neuronal vulnerability to ELS. Instead, regional levels of maturation might need to be taken into account (Lopez-Meraz et al., 2010). Accumulating evidence suggests that development is not necessarily synchronized among distinct cortical areas, but that structural and functional maturation progress in a caudal-to-rostral direction (Huttenlocher, 1990; Huttenlocher and Dabholkar, 1997; Kurth et al., 2010; Bianchi et al., 2013). In agreement, we have recently shown that endogenous Up state activity matures faster in S1 compared to M1 in the developing mouse (Rigas et al., 2015). In addition, physiological and anatomical findings also show that maturational levels are not uniform throughout the rat hippocampus, with CA1 maturing earlier than the dentate gyrus (Harris and Teyler, 1984; Bekenstein and Lothman, 1991a,b; Lowenstein and Alldredge, 1998). Therefore, one could expect distinct cortical regions to respond differently to a common seizure experience depending on their maturational stage.

### The Effects of ELS Depend on Seizure Frequency and Pattern

While the impact of recurrent seizures has been addressed experimentally (Stafstrom et al., 1992; Huang et al., 2002; Lai et al., 2002; Riviello et al., 2002; Karnam et al., 2009a,b), to our knowledge the direct comparison of their effect to that of either an individual seizure (i.e., addressing the issue of *seizure frequency*) or of a single prolonged seizure whose duration would equal the total duration of multiple seizures (i.e., addressing the issue of *seizure pattern*), has received scarce or no attention, respectively (Ni et al., 2005). Both issues are inherently associated with the on-going dilemma that neurologists face in the clinic on whether or when seizures should be treated or not. For example, it is debated whether early single or non-persistent seizures should be treated (Hughes, 2010). The dilemma rises from the fact that it is unclear whether the cognitive impairments often associated with early-seizures are due to the seizures *per se*, or are rather a consequence of either the underlying etiology of seizures, or of the antiepileptic drug therapy itself (Reeta et al., 2009, 2010). Hence, the relative merit of treating early-life non-persistent seizures with anti-epileptic drugs over leaving them untreated is currently not well understood.

As opposed to clinical observations or other experimental models in which seizure durations are difficult to control, in our study we were able to induce seizures in a well-regulated manner and thus test the effect of both seizure frequency and of seizure pattern. We found (a) a pattern effect of ELS: a single 60 min event of status epilepticus, but not multiple seizures of *equal total duration*, suffices to cause a persistent change in M1 and S1 network excitability in the mature brain (Figure 4). And (b) a frequency effect of ELS: multiple, but not single seizures during early life lead to a significant increase in the theta power of S1 network activity during adulthood (Figure 6). Hence, our results suggest that short duration seizures recurring over a brief period in early life may be more benign compared to even a single event of status epilepticus, in line with clinical observations for febrile seizures (Annegers et al., 1987; French et al., 1993; Verity et al., 1998). In addition, our findings are in agreement to data from other experimental models showing the developing brain to be resistant to single seizures (Albala et al., 1984; Nitecka et al., 1984; Sperber et al., 1991; Thurber et al., 1992; Liu et al., 1994; Sarkisian et al., 1997; Riviello et al., 2002), but differ from them since we show a significant effect of recurring ELS (Figure 6).

### The Long-Term Effects of ELS Depend on the Age at Which They Occur

Our data indicate that recurrent early seizures caused changes in the spectral content of spontaneous Up states in the S1 cortex of the adult brain (increase in theta power). This effect was both age- and region-specific since it occurred in the older but not the younger age group (P19–23 vs. P9–15) and in S1 but not M1 cortex (Figure 5). In a recent study of ours we traced changes in the spectral content of spontaneous Up states of the mouse S1 cortex during development and maturation. Interestingly, we found that the most prominent changes take place during the transition from the second to the third postnatal week: the power of lower frequencies (delta+theta) decrease and the power of higher frequencies (beta+gamma) increase (Rigas et al., 2015). We could therefore speculate that the age-dependent long-term effects of ELS on theta may rise from the fact that seizures act on uneven levels of theta activity (i.e., higher at P9–15 vs. lower at P19–23). In addition, this age-specific effect was cortex-dependent since only S1 cortex was affected. Our previous work has shown that the developmental trajectory of endogenous network dynamics is faster in S1 compared to M1 cortex, with the peak of Up state activity occurring at P19 vs. P30, in S1 vs. M1 cortex, respectively (Rigas et al., 2015). Therefore multiple ELS during P19-23 would act on distinct levels of network excitability in the two areas (higher in S1 and lower in M1), which in turn could contribute to the increased effect on the spectral content in the adult S1 but not M1 cortex.

The age-specific effect of ELS that we found is in agreement with several lines of evidence, both clinical and experimental, supporting a higher vulnerability of the older than the younger brain to seizures. In particular, clinical evidence suggests that status epilepticus (SE) in young children leads to lower mortality and better cognitive outcomes when compared to SE in adults and the elderly (Maytal et al., 1989; Lowenstein and Alldredge, 1998; Leppik et al., 2006; Towne, 2007). For example, many studies show that prolonged seizures are less likely to result to neuronal loss or synaptic rearrangement in the brain of infants and children compared to the mature brain (Sperber et al., 1992; Holmes and Ben-Ari, 2001; Bender et al., 2003; Porter et al., 2004; Baram et al., 2011). Similarly, animal studies of experimental seizures have shown the immature hippocampus to be more resilient to seizure-induced neuronal cell death and synaptic reorganization (Sperber et al., 1991, 1999; Lado et al., 2000; Sperber and Moshe, 2001; Riviello et al., 2002). In the current study, however, we also wanted to explore whether the long term outcome of ELS in the immature brain differed depending on the specific *developmental stage* these occurred. To this end we tested developing mice at two ages: P9–15 and P19–23. Given that rodents are born prematurely compared to humans and that mice sexually mature around P30–35 (Safranski et al., 1993), the ages that we studied are equivalent to human infants and prepubertal children (Nehlig, 1997; Velisek and Moshe, 2002; Sengupta, 2013; Dutta and Sengupta, 2016). While the differential effects of seizures on the immature and adult brain are well established, less is known on whether and how the effects of ELS may differ depending on when they occur during development. For example, resistance of the human brain to seizures differs among developmental stages, gradually decreasing from infancy to childhood and adolescence as recently reviewed (Nickels, 2015). Respectively, animal studies have shown that the effects of ELS in rats differ depending on whether they occur before or after P20, with younger rats being more resilient (Stafstrom et al., 1992; Sayin et al., 2015). In addition, recent research has indicated the days P20–30 as a critical period for the long-term outcome of ELS, at least for the rat hippocampus (Sayin et al., 2015). Our results extend these studies and indicate that the broad divisions “mature vs. immature,” or “adult vs. developing” are not adequate enough to explore differential brain vulnerability to seizures. Instead, this issue should be examined at a higher temporal resolution by examining specific and more restricted periods during development and maturation.

### Significance and Perspectives

The reported electrophysiological results consist the first, to our knowledge, evidence that seizures during early development may cause permanent changes in the local network dynamics of the adult neocortex. We therefore propose that spontaneous cortical network activity, in the form of recurring Up states, may serve as a neurophysiological measure to describe and study the long-term effects of ELS on cortical function and excitability, in both the lab and the clinic. The current results are part of a larger ongoing study that includes behavioral assessment as well as evaluation of brain cytoarchitecture in order to corroborate electrophysiological findings and explore the underlying mechanisms responsible for the differential effects.

Cortical neurons form recurrent networks which synchronize individual cells and are intrinsically active in the form of oscillating activity, visible at increasingly macroscopic neurophysiological levels: from single cells to LFPs; to the clinically relevant electroencephalography (EEG). Synchronized oscillating neuronal networks are viewed as the “middle ground” between single-neuron activity and behavior (Buzsaki and Draguhn, 2004). Although, research of experimental seizures has provided invaluable insights to the cellular and synaptic changes that ELS can cause to the adult cortex (as reviewed in the *Introduction*), whether, to what extent and how these changes actually contribute to higher levels of organization such as the *neuronal network*, as a *final functional pathway* that defines brain physiology and ultimately behavior, are issues that have received less attention. Our results support the idea that spontaneous Up states may provide the necessary framework to link molecular, cellular and synaptic changes to ELS-induced local network dynamics. Importantly, since this activity is present not only in the intact brain but also at the reduced level of the cortical slice, it also provides researchers with a useful experimental tool with which to explore the underlying cellular and synaptic mechanisms.

Spontaneous Up and Down states are the intracellular correlates of the *slow oscillation*, the electroencephalographic hallmark of quiescent states of the brain, such as non-REM sleep, anesthesia and quiet wakefulness (Steriade et al., 1993; Crochet and Petersen, 2006). It is noteworthy that *in vivo* recordings of spontaneous network cortical activity during rest are currently employed in the clinic for the discovery of biomarkers of psychiatric disorders (Wada et al., 1998; Kissler et al., 2000; Sorg et al., 2007; Gandal et al., 2010), while EEG recordings during sleep have been used to describe cortical development in humans (Buchmann et al., 2011). If replicated *in vivo*, the results of our study raise the interesting possibility that the parameters of the slow oscillation in EEG recordings may provide clinicians and researchers with *endophenotypes* of seizure-induced cortical malfunctions. By definition, an endophenotype is the biological manifestation of a disease at a *reduced* level of biological organization as opposed to the macro-level of behavior (Gottesman and Shields, 1973; Almasy and Blangero, 2001; Gottesman and Gould, 2003; Hasler et al., 2004; Gould and Gottesman, 2006). Thus, in order for biological research of mental disorders to proceed, it is essential to ‘decompose’ the disorder into simpler parameters that can serve as endophenotypes. In this perspective, studying the activity of local cortical microcircuits may provide useful insights toward understanding the brain pathology induced by ELS.

## Author Contributions

PR contributed to the conception and design of the work; the acquisition, analysis, and interpretation of data; and drafting the manuscript and critically revising it. CS contributed to the acquisition of the data and critically revising the manuscript. MN, CP, AspK, and AnnKatri contributed to experiments of seizure induction. AniK contributed to data analysis and drafting the manuscript. LL contributed to data analysis, drafting the manuscript and critically revising it. KA and CZ contributed to data analysis. IS contributed to the conception and design of the work, interpretation of data, and critically revising the manuscript.

## Conflict of Interest Statement

The authors declare that the research was conducted in the absence of any commercial or financial relationships that could be construed as a potential conflict of interest.

## Footnotes

1 http://depts.washington.edu/aimgroup/proj/art/
