# Supplementary material for: Long-Term Effects of Early Life Seizures on Endogenous Local Network Activity of the Mouse Neocortex
Source: Front Synaptic Neurosci. 2018 Nov 27;10:43. doi: 10.3389/fnsyn.2018.00043 (PMC6277496; doi:10.3389/fnsyn.2018.00043)
Supplement: Supplementary file 1 [file Data_Sheet_1.pdf]

In this Supplementary Text we present in their full extent the results of our factorial analysis of variance (ANOVA) for multiple group comparisons regarding five (5) sets of analysis, namely:

(1) The long-term effects of multiple early-life administrations of diazepam (DZP) on spontaneous cortical Up states
(2) The long-term effects of early-life status epilepticus (SE) on local cortical network activity are region-specific
(3) The long-term effects of ELS on local cortical network activity depend on the duration of individual seizures, rather than on their frequency
(4) Younger ages are more resilient to the long-term effects of ELS on local cortical network activity
(5) The long-term effects of ELS on cortical dynamics depend on their frequency

Each set of analysis is presented as a distinct section and in each case the strategy that we followed can be briefly described by the following steps:

(i) ***Testing normality***. Data of all Up states variables (*occurrence, duration, amplitude, rectified area, network index, normalized delta, normalized theta, normalized alpha, normalized beta and normalized gamma*) within each group as defined by the combination of factors we were interested each time were tested for normality using the Shapiro-Wilk test. We considered data to be normally distributed when $p>0.05$ and non-normally distributed when $p<0.05$. These p values are described in the first table of each section. The second table of each section describes the data within each group as their mean ± standard deviation (sd) as well as their median and interquartile range. The former pertains to normally distributed data and the latter to non-normally distributed data as concluded based on the preceding table with the results (p-values) of normality tests.

(ii) ***Transforming data***. In the perspective of applying for each Up state variable a factorial analysis of variance (ANOVA) for multiple groups comparisons it was often the case that data for the same Up state variable would be normally distributed in one group but not in another group we were interested in comparing. In such cases data from all groups (either normally or non-normally distributed) of the specific Up state variable were transformed according to the rules of the Aligned Rank Transformation (ART) (Wobbrock et al. 2011) using the ARTool software (http://depts.washington.edu/aimgroup/proj/art/). Data transformation of a specific Up state variable is designated by the prefix ART, for example *ARToccurrence* means that the Up state variable *occurrence* was transformed according to ART.

(iii) ***Testing the significance of interactions***. Interactions of all factors of interest were tested in order: from higher to lower levels (i.e. numbers of factors) when the former were non-significant. For example in a three way ANOVA we would first test the three-way interaction. If it was not significant we would proceed with two-way interactions and, in turn, if those were also not significant we would test the factors' *main effects*. It should be noted that in such a case as we would proceed tests from higher to lower levels of interactions, normality tests of data would be performed again at each level since it would involve a different combination of factors and data. Respectively, data would also be ART transformed again according to the combination of factors of interest. Therefore, ART transformed data was also defined by a suffix that indicated the specific factors that were combined for each set of data and whose interaction we were interested in tested. Extending our previous example *ARToccurrenceCortexXSeizures* means that the *occurrence* of Up states was ART transformed for the interaction of *Cortex* and *Seizures*.

(iv) ***Testing simple effects***. On the contrary, if an interaction is significant we ignored any other lower-level interactions or main effects and proceeded with posthoc analysis of *simple effects*. Here, we would ART transform data again for those factors whose simple effects are not being tested, within each level of the factor whose simple effect is being tested. For example if there is a significant interaction of three factors (cortex, seizures and age) for ART transformed Up state occurrence (*ARTOccurrenceCortexXSeizuresXAge*) and we are interested in testing the simple effects of age, the data would be transformed in respect to the *CortexXSeizures* interaction within each level of the factor *Age* and this anew transformation would be designated as *ARTOccurrenceCortexXSeizuresBYAge.*

1. The long-term effects of multiple early-life administrations of diazepam (DZP) on spontaneous cortical Up states

Since all PTZ-treated but none saline-treated (control) mice were injected with the anticonvulsant diazepam (DZP, 2mg/kg) in order to end seizures (*2.3. Standardization of seizure induction protocol*), we asked whether early life administrations of DZP (2mg/kg) *per se* may affect cortical network dynamics in the long run, in the adult brain. To answer this question we compared spontaneous Up states in the adult S1 and M1 cortex of mice that had received three administrations of DZP (2mg/kg) on every second day at P19-23 to those of age-matched saline-injected mice (*saline:* $N_{animals}=6$, $N_{slices}=11$ *(S1), 7(M1); DZP:* $N_{animals}=6$, $N_{slices}=11$ *(S1), 5(M1)*). To this end we conducted a two-way ANOVA of the effect of two independent variables: injections (saline and DZP) and type of cortex (S1 and M1) on Up states dynamics as quantified by respective parameters. We didn’t find a significant interaction of these two factors for any Up state measurement (*Supplementary Text, Section 1.1.2, Table 3*). Moreover, analysis of the main effects of DZP (*injections*) on spontaneous Up states did not reveal any significant effect (*Supplementary Text, Section 1.2.2, Table 6*).

*1.1. Analysis of two-way interaction: cortex * injections*

*1.1.1. Normality tests of data distribution and descriptives of data for each group (cortex * injections)*

		S1 cortex			M1 cortex		
		Shapiro-Wilk			Shapiro-Wilk		
	Injections	Statistic	df	Sig.	Statistic	df	Sig.
Occurrence	saline	.831	11	.024	.926	7	.514
	DZP	.904	11	.204	.771	5	.046
Duration	saline	.836	11	.028	.975	7	.934
	DZP	.949	11	.625	.846	5	.182
Amplitude	saline	.957	11	.737	.937	7	.609
	DZP	.823	11	.019	.952	5	.748
RectifiedArea	saline	.957	11	.728	.926	7	.516
	DZP	.858	11	.054	.862	5	.234
Up state Index	saline	.875	11	.090	.819	7	.063
	DZP	.802	11	.010	.806	5	.090
NormalizedDelta	saline	.885	11	.121	.912	7	.412
	DZP	.975	11	.934	.985	5	.959
NormalizedTheta	saline	.953	11	.683	.862	7	.158
	DZP	.819	11	.017	.836	5	.154
NormalizedAlpha	saline	.935	11	0.46	.880	7	.226
	DZP	.971	11	0.9	.942	5	.680
NormalizedBeta	saline	.933	11	.438	.598	7	.000
	DZP	.820	11	.017	.877	5	.297
NormalizedGamma	saline	.947	11	.603	.782	7	.027
	DZP	.938	11	.501	.960	5	.807

Table 1. Output (significance) of Shapiro-Wilki's normality tests of distribution of data within each group. Data is color-coded darker colors indicating lower p values.

		S1 cortex				M1 cortex			
	Injections	Mean	SD	Median	IQR	Mean	SD	Median	IQR
Occurrence	saline	0.93	0.72	0.61	0.90	0.69	0.35	0.65	0.65
	DZP	0.78	0.41	0.66	0.50	1.11	0.94	0.58	1.79
Duration	saline	1.53	0.38	1.67	0.50	1.67	0.50	1.75	0.82
	DZP	1.50	0.36	1.52	0.61	1.69	0.24	1.67	0.48
Amplitude	saline	-80.09	36.39	-79.58	63.71	-81.73	30.11	-77.54	50.33
	DZP	-84.59	31.14	-76.45	34.02	-77.27	19.17	-71.94	36.29
RectifiedArea	saline	0.11	0.04	0.11	0.09	0.12	0.05	0.12	0.08
	DZP	0.11	0.04	0.09	0.08	0.12	0.03	0.13	0.06
Up state Index	saline	0.12	0.11	0.09	0.15	0.09	0.09	0.06	0.11
	DZP	0.09	0.08	0.09	0.08	0.12	0.10	0.06	0.18
NormalizedDelta	saline	0.42	0.08	0.40	0.14	0.38	0.11	0.41	0.18
	DZP	0.41	0.05	0.41	0.10	0.38	0.06	0.37	0.10
NormalizedTheta	saline	0.15	0.05	0.15	0.05	0.09	0.03	0.09	0.02
	DZP	0.14	0.04	0.13	0.05	0.12	0.04	0.11	0.05
NormalizedAlpha	saline	0.06	0.02	0.06	0.02	0.04	0.02	0.04	0.02
	DZP	0.06	0.01	0.06	0.02	0.05	0.02	0.04	0.03
NormalizedBeta	saline	0.08	0.04	0.09	0.06	0.09	0.09	0.06	0.03
	DZP	0.09	0.04	0.08	0.04	0.10	0.05	0.09	0.09
NormalizedGamma	saline	0.07	0.04	0.06	0.06	0.11	0.10	0.06	0.09
	DZP	0.08	0.04	0.08	0.04	0.11	0.03	0.11	0.06

Table 2. Data of all ten (10) parameters of spontaneous Up states per each experimental group presented as mean and standard deviation (SD) as well as median and interquartile range (IQR). Although mean ± sd and median (IQR) are used to describe mainly normally and non-normally distributed data, respectively, we have included both descriptives for each parameter. The normality of distribution of data for each group can be seen in preceding table 1.

1.1.2. Analysis of two-way interaction

Two-way ANOVA: injections * cortex

Between Subjects Factors

Factor	Level	N
Injections	Saline	18
	DZP	16
Cortex	S1	22
	M1	12

Source	Dependent Variable	Type III Sum of Squares	df	Mean Square	F	Sig.
Injections * Cortex	ARTOccurrenceCortexXInjections	1.704	1	1.704	.016	.901
	ARTDurationCortexXInjections	15.082	1	15.082	.139	.712
	ARTAmplitudeCortexXInjections	6.984	1	6.984	.064	.802
	RectifiedArea	.000	1	.000	.088	.769
	ART Up state IndexCortexXInjections	40.148	1	40.148	.376	.544
	NormalizedDelta	.000	1	.000	.025	.875
	ARTThetaCortexXInjections	310.371	1	310.371	3.168	.085
	NormalizedAlpha	.000	1	.000	.253	.619
	ARTBetaCortexXInjections	95.062	1	95.062	.971	.332
	ARTGammaCortexXInjections	5.023	1	5.023	.048	.828
Error	ARTOccurrenceCortexXInjections	3262.382	30	108.746		
	ARTDurationCortexXInjections	3249.592	30	108.320		
	ARTAmplitudeCortexXInjections	3258.592	30	108.620		
	RectifiedArea	.060	30	.002		
	ART Up state IndexCortexXInjections	3200.787	30	106.693		
	NormalizedDelta	.177	30	.006		
	ARTThetaCortexXInjections	2939.066	30	97.969		
	NormalizedAlpha	.010	30	.000		
	ARTBetaCortexXInjections	2935.732	30	97.858		
	ARTGammaCortexXInjections	3152.701	30	105.090		

Table 3. The significance of interaction between the effects of injections and cortex for all ten (10) parameters of spontaneous Up states. Data that were not normally distributed (Table 1) were previously transformed according to the Aligned Rank Transformation (ART) rules.

1.2. Analysis of main effect of injections

1.2.1. Normality tests of data distribution and descriptives of data for each group (injections: DZP, saline)

		Shapiro-Wilk		
	Injections	Statistic	df	Sig.
Occurrence	saline	.826	18	.004
	DZP	.834	16	.008
Duration	saline	.947	18	.375
	DZP	.967	16	.780
Amplitude	saline	.960	18	.610
	DZP	.841	16	.010
RectifiedArea	saline	.970	18	.793
	DZP	.886	16	.047
Up state Index	saline	.852	18	.009
	DZP	.818	16	.005
NormalizedDelta	saline	.977	18	.909
	DZP	.987	16	.996
NormalizedTheta	saline	.931	18	.206
	DZP	.865	16	.023
NormalizedAlpha	saline	.947	18	.381
	DZP	.987	16	.995
NormalizedBeta	saline	.713	18	.000
	DZP	.867	16	.024
NormalizedGamma	saline	.770	18	.001
	DZP	.964	16	.727

Table 4. Output (significance) of Shapiro-Wilki's normality tests of distribution of data within each group. Data is color-coded darker colors indicating lower p values.

	Injections	Mean	SD	Median	IQR
Occurrence	saline	0.84	0.60	0.63	0.72
	DZP	0.88	0.61	0.65	0.69
Duration	saline	1.58	0.42	1.71	0.47
	DZP	1.56	0.33	1.55	0.64
Amplitude	saline	-80.73	33.16	-78.56	59.51
	DZP	-82.30	27.51	-74.20	32.44
RectifiedArea	saline	0.11	0.05	0.11	0.08
	DZP	0.11	0.04	0.11	0.08
Up state Index	saline	0.11	0.10	0.07	0.13
	DZP	0.10	0.08	0.07	0.09
NormalizedDelta	saline	0.41	0.09	0.40	0.15
	DZP	0.40	0.05	0.40	0.09
NormalizedTheta	saline	0.13	0.05	0.13	0.08
	DZP	0.13	0.04	0.12	0.05
NormalizedAlpha	saline	0.05	0.02	0.05	0.03
	DZP	0.05	0.02	0.05	0.02
NormalizedBeta	saline	0.09	0.06	0.07	0.05
	DZP	0.09	0.04	0.09	0.04
NormalizedGamma	saline	0.09	0.07	0.06	0.07
	DZP	0.09	0.04	0.09	0.05

Table 5. Data of all ten (10) parameters of spontaneous Up states per each experimental group presented as mean and standard deviation (SD) as well as median and interquartile range (IQR). Although mean ± sd and median (IQR) are used to describe mainly normally and non-normally distributed data, respectively, we have included both descriptives for each parameter. The normality of distribution of data for each group can be seen in the preceding table 4.

1.2.2. Main effects of injections

One-way ANOVA: injections

Between Subjects Factors

Factor	Level	N
Injections	Saline	18
	DZP	16

Source	Dependent Variable	Type III Sum of Squares	df	Mean Square	F	Sig.
Injections	ARTOccurrenceBYInjections	13.016	1	13.016	.128	.723
	Duration	.007	1	.007	.046	.832
	ARTAmplitudeBYInjections	1.063	1	1.063	.010	.919
	ARTRectifiedAreaBYInjections	17.000	1	17.000	.169	.684
	ART Up state BYInjections	15.613	1	15.613	.154	.697
	NormalizedDelta	.000	1	.000	.046	.831
	ARTThetaBYInjections	40.405	1	40.405	.403	.530
	NormalizedAlpha	.000	1	.000	.054	.818
	ARTBetaBYInjections	113.451	1	113.451	1.167	.288
	ARTGammaBYInjections	144.618	1	144.618	1.490	.231
Error	ARTOccurrenceBYInjections	3257.484	32	101.796		
	Duration	4.672	32	.146		
	ARTAmplitudeBYInjections	3270.938	32	102.217		
	ARTRectifiedAreaBYInjections	3221.500	32	100.672		
	ART Up state BYInjections	3236.387	32	101.137		
	NormalizedDelta	.186	32	.006		
	ARTThetaBYInjections	3206.595	32	100.206		
	NormalizedAlpha	.012	32	.000		
	ARTBetaBYInjections	3110.049	32	97.189		
	ARTGammaBYInjections	3106.382	32	97.074		

Table 6. The significance of the main effect of *injections* on all ten (10) parameters of spontaneous Up states. Data that were not normally distributed (Table 4) were previously transformed according to the Aligned Rank Transformation (ART) rules.

2. The long-term effects of ELS on local cortical network activity are region-specific

2.1. *Analysis of two-way interaction: cortex * injections*

2.1.1. *Normality tests of data distribution and descriptive of data for each group (cortex * injections)*

		S1 cortex			M1 cortex		
		Shapiro-Wilk			Shapiro-Wilk		
	Injections	Statistic	df	Sig.	Statistic	df	Sig.
Occurrence	saline	.867	12	.060	.815	10	.022
	PTZ	.796	14	.004	.928	10	.433
Duration	saline	.927	12	.348	.969	10	.884
	PTZ	.901	14	.116	.901	10	.223
Amplitude	saline	.918	12	.267	.925	10	.399
	PTZ	.894	14	.091	.765	10	.005
RectifiedArea	saline	.898	12	.151	.974	10	.926
	PTZ	.895	14	.095	.931	10	.461
Up state Index	saline	.895	12	.135	.763	10	.005
	PTZ	.712	14	.000	.951	10	.683
NormalizedDelta	saline	.783	12	.006	.887	10	.157
	PTZ	.951	14	.576	.969	10	.877
NormalizedTheta	saline	.897	12	.144	.985	10	.986
	PTZ	.915	14	.188	.931	10	.458
NormalizedAlpha	saline	.522	12	.000	.972	10	.908
	PTZ	.916	14	.190	.917	10	.330
NormalizedBeta	saline	.925	12	.329	.797	10	.013
	PTZ	.904	14	.129	.829	10	.033
NormalizedGamma	saline	.952	12	.674	.933	10	.481
	PTZ	.966	14	.820	.897	10	.202

Table 7. Output (significance) of Shapiro-Wilki's normality tests of distribution of data within each group. Data is color-coded darker colors indicating lower p values.

		S1 cortex				M1 cortex			
	Injections	Mean	SD	Median	IQR	Mean	SD	Median	IQR
Occurrence	saline	0.97	0.58	0.80	1.01	0.58	0.40	0.36	0.74
	PTZ	0.75	0.62	0.52	0.64	1.14	0.56	1.14	1.14
Duration	saline	1.35	0.23	1.35	0.23	1.50	0.43	1.51	0.70
	PTZ	1.42	0.33	1.44	0.69	1.82	0.58	1.59	0.90
Amplitude	saline	-64.83	24.78	-65.00	33.00	-49.70	12.31	-51.00	23.25
	PTZ	-54.64	20.68	-51.00	31.50	-64.00	18.18	-62.50	10.25
RectifiedArea	saline	0.09	0.04	0.08	0.05	0.08	0.04	0.09	0.05
	PTZ	0.08	0.04	0.08	0.06	0.12	0.04	0.13	0.08
Up state Index	saline	0.07	0.02	0.08	0.04	0.04	0.03	0.03	0.03
	PTZ	0.07	0.08	0.05	0.06	0.14	0.09	0.12	0.15
NormalizedDelta	saline	0.40	0.14	0.41	0.10	0.38	0.08	0.38	0.08
	PTZ	0.42	0.06	0.41	0.10	0.40	0.06	0.40	0.06
NormalizedTheta	saline	0.14	0.04	0.14	0.06	0.13	0.04	0.14	0.06
	PTZ	0.14	0.04	0.14	0.06	0.10	0.02	0.11	0.03
NormalizedAlpha	saline	0.07	0.04	0.06	0.01	0.06	0.02	0.06	0.04
	PTZ	0.06	0.01	0.06	0.02	0.04	0.01	0.04	0.03
NormalizedBeta	saline	0.09	0.03	0.09	0.04	0.11	0.06	0.12	0.07
	PTZ	0.09	0.03	0.09	0.04	0.08	0.04	0.08	0.02
NormalizedGamma	saline	0.09	0.05	0.09	0.07	0.09	0.04	0.09	0.06
	PTZ	0.10	0.04	0.10	0.07	0.09	0.04	0.09	0.03

Table 8. Data of all ten (10) parameters of spontaneous Up states per each experimental group presented as mean and standard deviation (SD) as well as median and interquartile range (IQR). Although mean ± sd and median (IQR) are used to describe mainly normally and non-normally distributed data, respectively, we have included both descriptives for each parameter. The normality of distribution of data for each group can be seen in the preceding table 7.

2.1.2. *Analysis of two-way interaction*

Two-way ANOVA: seizures * cortex

Between Subjects Factors

Factor	Level	N
Injections	Saline	22
	PTZ	24
Cortex	S1	26
	M1	20

Source	Dependent variable	Sum of Squares	df	Mean Square	F	Sig.
Injections *Cortex	ARTOccurrenceCortexXInjections	1436.391	1	1436.391	9.071	0.004
	Duration	.181	1	0.181	1.13	0.294
	ARTAmplitudeCortexXInjections	731.308	1	731.308	4.172	0.047
	RectifiedArea	.006	1	0.006	4.151	0.048
	ART Up state IndexCortexXInjections	1827.447	1	1827.447	12.7	0.001
	ARTNormalizedDeltaCortexXInjections	3.515	1	3.515	0.019	0.892
	NormalizedTheta	.002	1	0.002	1.524	0.224
	ARTNormalizedAlphaCortexXInjections	494.519	1	494.519	2.97	0.092
	ARTNormalizedBetaCortexXInjections	260.038	1	260.038	1.405	0.243
	NormalizedGamma	.000	1	0	0.169	0.683
Error	ARTOccurrenceCortexXInjections	6650.431	42	158.344		
	Duration	6.738	42	.160		
	ARTAmplitudeCortexXInjections	7362.664	42	175.302		
	RectifiedArea	0.063	42	.002		
	ART Up state IndexCortexXInjections	6043.595	42	143.895		
	ARTNormalizedDeltaCortexXInjections	7920.917	42	188.593		
	NormalizedTheta	0.059	42	.001		
	ARTNormalizedAlphaCortexXInjections	6992.845	42	166.496		
	ARTNormalizedBetaCortexXInjections	7776.095	42	185.145		
	NormalizedGamma	0.073	42	.002		

Table 9. The significance of interaction between the effects of seizures and cortex for all ten (10) parameters of spontaneous Up states. Data that were not normally distributed (Table 7) were previously transformed according to the Aligned Rank Transformation (ART) rules.

2.2. Simple effects analysis

2.2.1. Simple effects of seizures

2.2.1.1. Simple effect of seizures on Occurrence

Estimated Marginal Means

Cortex * Injections

Estimates

Dependent Variable:ARTOccurrenceBYcortex

Cortex	Injections	Mean	Std. Error	95% Confidence Interval Lower Bound	95% Confidence Interval Upper Bound
S1	saline	15.708	1.902	11.869	19.548
	PTZ	11.607	1.761	8.053	15.162
M1	saline	7.400	2.084	3.194	11.606
	PTZ	13.600	2.084	9.394	17.806

Pairwise Comparisons

Dependent Variable:ARTOccurrenceBYcortex

Cortex	(I) Injections	(J) Injections	Mean Difference (I-J)	Std. Error	Sig.[a]	95% Confidence Interval for Difference[a] Lower Bound	95% Confidence Interval for Difference[a] Upper Bound
S1	saline	PTZ	4.101	2.593	.121	-1.131	9.333
	PTZ	saline	-4.101	2.593	.121	-9.333	1.131
M1	saline	PTZ	-6.200*	2.947	.041	-12.148	-.252
	PTZ	saline	6.200*	2.947	.041	.252	12.148

Based on estimated marginal means

a. Adjustment for multiple comparisons: Bonferroni.

*. The mean difference is significant at the .05 level.

Univariate Tests

Dependent Variable:ARTOccurrenceBYcortex

Cortex		Sum of Squares	df	Mean Square	F	Sig.
S1	Contrast	108.682	1	108.682	2.502	.121
	Error	1824.118	42	43.431		
M1	Contrast	192.200	1	192.200	4.425	**.041**
	Error	1824.118	42	43.431		

Each F tests the simple effects of Injections within each level combination of the other effects shown. These tests are based on the linearly independent pairwise comparisons among the estimated marginal means.

2.2.1.2.Simple effect of seizures on Up state Index

Estimated Marginal Means

Injections * Cortex

Estimates

Dependent Variable:ART Up state IndexInjectionsBYCortex

Injections	Cortex	Mean	Std. Error	95% Confidence Interval	
				Lower Bound	Upper Bound
saline	S1	16.583	1.779	12.992	20.174
	M1	6.700	1.949	2.766	10.634
PTZ	S1	10.857	1.647	7.533	14.182
	M1	14.300	1.949	10.366	18.234

Pairwise Comparisons

Dependent Variable:ART Up state IndexInjectionsBYCortex

Cortex	(I) Injections	(J) Injections	Mean Difference (I-J)	Std. Error	Sig.[a]	95% Confidence Interval for Difference[a]	
						Lower Bound	Upper Bound
S1	saline	PTZ	5.726*	2.425	.023	.832	10.620
	PTZ	saline	-5.726*	2.425	.023	-10.620	-.832
M1	saline	PTZ	-7.600*	2.757	.009	-13.163	-2.037
	PTZ	saline	7.600*	2.757	.009	2.037	13.163

Based on estimated marginal means

*. The mean difference is significant at the .05 level.

a. Adjustment for multiple comparisons: Bonferroni.

Univariate Tests

Dependent Variable:ART Up state IndexInjectionsBYCortex

Cortex		Sum of Squares	df	Mean Square	F	Sig.
S1	Contrast	211.869	1	211.869	5.576	**.023**
	Error	1595.831	42	37.996		
M1	Contrast	288.800	1	288.800	7.601	**.009**
	Error	1595.831	42	37.996		

Each F tests the simple effects of Injections within each level combination of the other effects shown. These tests are based on the linearly independent pairwise comparisons among the estimated marginal means.

2.2.1.3.Simple effect of seizures on Amplitude

Estimated Marginal Means

Injections * Cortex

Estimates

Dependent Variable:ARTAmplitudeBYcortex

Injections	Cortex	Mean	Std. Error	95% Confidence Interval	
				Lower Bound	Upper Bound
saline	S1	11.458	1.942	7.538	15.378
	M1	13.000	2.128	8.706	17.294
PTZ	S1	15.250	1.798	11.621	18.879
	M1	8.000	2.128	3.706	12.294

Pairwise Comparisons

Dependent Variable:ARTAmplitudeBYcortex

Cortex	(I) Injections	(J) Injections	Mean Difference (I-J)	Std. Error	Sig.[a]	95% Confidence Interval for Difference[a]	
						Lower Bound	Upper Bound
S1	saline	PTZ	-3.792	2.647	.159	-9.134	1.550
	PTZ	saline	3.792	2.647	.159	-1.550	9.134
M1	saline	PTZ	5.000	3.009	.104	-1.073	11.073
	PTZ	saline	-5.000	3.009	.104	-11.073	1.073

Based on estimated marginal means

a. Adjustment for multiple comparisons: Bonferroni.

Univariate Tests

Dependent Variable:ARTAmplitudeBYcortex

Cortex		Sum of Squares	df	Mean Square	F	Sig.
S1	Contrast	92.896	1	92.896	2.052	.159
	Error	1901.604	42	45.276		
M1	Contrast	125.000	1	125.000	2.761	.104
	Error	1901.604	42	45.276		

Each F tests the simple effects of Injections within each level combination of the other effects shown. These tests are based on the linearly independent pairwise comparisons among the estimated marginal means.

2.2.1.4. Simple effect of seizures on Rectified Area

Estimated Marginal Means

Injections * Cortex

Estimates

Dependent Variable:RectifiedArea

Injections	Cortex	Mean	Std. Error	95% Confidence Interval	
				Lower Bound	Upper Bound
saline	S1	.090	.011	.068	.113
	M1	.084	.012	.059	.108
PTZ	S1	.079	.010	.058	.100
	M1	.119	.012	.094	.144

Pairwise Comparisons

Dependent Variable:RectifiedArea

Cortex	(I) Injections	(J) Injections	Mean Difference (I-J)	Std. Error	Sig.[a]	95% Confidence Interval for Difference[a]	
						Lower Bound	Upper Bound
S1	saline	PTZ	.012	.015	.450	-.019	.042
	PTZ	saline	-.012	.015	.450	-.042	.019
M1	saline	PTZ	-.035*	.017	.047	-.070	.000
	PTZ	saline	.035*	.017	.047	.000	.070

Based on estimated marginal means

a. Adjustment for multiple comparisons: Bonferroni.

*. The mean difference is significant at the .05 level.

Univariate Tests

Dependent Variable:RectifiedArea

Cortex		Sum of Squares	df	Mean Square	F	Sig.
S1	Contrast	.001	1	.001	.582	.450
	Error	.063	42	.002		
M1	Contrast	.006	1	.006	4.172	.047
	Error	.063	42	.002		

Each F tests the simple effects of Injections within each level combination of the other effects shown. These tests are based on the linearly independent pairwise comparisons among the estimated marginal means.

2.2.1.5.Simple effect of seizures on Up state Index

Estimated Marginal Means

Injections * Cortex

Estimates

Dependent Variable:ARTNetworkIndexInjectionsBYCortex

Injections	Cortex	Mean	Std. Error	95% Confidence Interval	
				Lower Bound	Upper Bound
saline	S1	16.583	1.779	12.992	20.174
	M1	6.700	1.949	2.766	10.634
PTZ	S1	10.857	1.647	7.533	14.182
	M1	14.300	1.949	10.366	18.234

Pairwise Comparisons

Dependent Variable:ART Up state IndexInjectionsBYCortex

Cortex	(I) Injections	(J) Injections	Mean Difference (I-J)	Std. Error	Sig.[a]	95% Confidence Interval for Difference[a]	
						Lower Bound	Upper Bound
S1	saline	PTZ	5.726*	2.425	.023	.832	10.620
	PTZ	saline	-5.726*	2.425	.023	-10.620	-.832
M1	saline	PTZ	-7.600*	2.757	.009	-13.163	-2.037
	PTZ	saline	7.600*	2.757	.009	2.037	13.163

Based on estimated marginal means

*. The mean difference is significant at the .05 level.

a. Adjustment for multiple comparisons: Bonferroni.

Univariate Tests

Dependent Variable:ART Up state IndexInjectionsBYCortex

Cortex		Sum of Squares	df	Mean Square	F	Sig.
S1	Contrast	211.869	1	211.869	5.576	**.023**
	Error	1595.831	42	37.996		
M1	Contrast	288.800	1	288.800	7.601	**.009**
	Error	1595.831	42	37.996		

Each F tests the simple effects of Injections within each level combination of the other effects shown. These tests are based on the linearly independent pairwise comparisons among the estimated marginal means.

2.3.2. Simple effects of Cortex

2.3.2.1. Simple effect of Cortex on Amplitude

In order to analyze the significant interactive effect of *Seizures* and *Cortex* on Up state amplitude we tested the simple effect of *Seizures* on Amplitude (*Section 2.2.1.3*), which however did not reveal any significant effect. We therefore proceeded with the analysis of the simple effects of *Cortex* on Amplitude.

Estimated Marginal Means

Injections * Cortex

Estimates

Dependent Variable:ARTAmplitudeBYInjections

Injections	Cortex	Mean	Std. Error	95% Confidence Interval Lower Bound	95% Confidence Interval Upper Bound
saline	S1	9.250	1.890	5.436	13.064
	M1	14.200	2.070	10.022	18.378
PTZ	S1	14.179	1.750	10.647	17.710
	M1	10.150	2.070	5.972	14.328

Pairwise Comparisons

Dependent Variable:ARTAmplitudeBYInjections

Injections	(I) Cortex	(J) Cortex	Mean Difference (I-J)	Std. Error	Sig.[a]	95% Confidence Interval for Difference[a] Lower Bound	95% Confidence Interval for Difference[a] Upper Bound
saline	S1	M1	-4.950	2.803	.085	-10.607	.707
	M1	S1	4.950	2.803	.085	-.707	10.607
PTZ	S1	M1	4.029	2.711	.145	-1.442	9.499
	M1	S1	-4.029	2.711	.145	-9.499	1.442

Based on estimated marginal means

a. Adjustment for multiple comparisons: Bonferroni.

Univariate Tests

Dependent Variable:ARTAmplitudeBYInjections

Injections		Sum of Squares	df	Mean Square	F	Sig.
saline	Contrast	133.650	1	133.650	3.118	.085
	Error	1800.179	42	42.861		
PTZ	Contrast	94.671	1	94.671	2.209	.145
	Error	1800.179	42	42.861		

Each F tests the simple effects of Cortex within each level combination of the other effects shown. These tests are based on the linearly independent pairwise comparisons among the estimated marginal means.

2.3. Analysis of main effects of seizures

Between Subjects Factors		
Factor	Level	N
Injections	Saline	22
	PTZ	24

Source	Dependent variable	Sum of Squares	df	Mean Square	F	Sig.
Injections	Duration	.337	1	.337	1.902	.175
	ARTNormalizedDelta	176.420	1	176.420	.979	.328
	NormalizedTheta	.002	1	.002	1.213	.277
	ARTNormalizedAlpha	.002	1	.002	2.889	.096
	ARTNormalizedBeta	.002	1	.002	1.521	.224
	NormalizedGamma	.000	1	.000	.007	.933
Error	Duration	7.795	44	.177		
	ARTNormalizedDelta	7925.080	44	180.115		
	NormalizedTheta	.067	44	.002		
	ARTNormalizedAlpha	.027	44	.001		
	ARTNormalizedBeta	.063	44	.001		
	NormalizedGamma	.074	44	.002		

Table 10. The significance of the main effect of seizures on spontaneous Up states.

3. The long-term effects of ELS on local cortical network activity depend on the duration of individual seizures, rather than on their frequency

*3.1. Analysis of three-way interaction: number of injections * cortex * injections*

*3.1.1. Normality tests of data distribution and descriptives of data for each group (number of injections * cortex * injections)*

		Number Of Injections = single						Number Of Injections = multiple					
		S1 cortex			M1 cortex			S1 cortex			M1 cortex		
		Shapiro-Wilk			Shapiro-Wilk			Shapiro-Wilk			Shapiro-Wilk		
	Injections	Statistic	df	Sig.	Statistic	df	Sig.	Statistic	df	Sig.	Statistic	df	Sig.
Occurrence	saline	.867	12	.060	.815	10	.022	.984	10	.982	.967	10	.866
	PTZ	.796	14	.004	.928	10	.433	.822	10	.027	.887	14	.073
Duration	saline	.927	12	.348	.969	10	.884	.959	10	.771	.936	10	.511
	PTZ	.901	14	.116	.901	10	.223	.942	10	.578	.953	14	.607
Amplitude	saline	.918	12	.267	.925	10	.399	.915	10	.316	.946	10	.623
	PTZ	.894	14	.091	.765	10	.005	.975	10	.931	.834	14	.014
RectifiedArea	saline	.898	12	.151	.974	10	.926	.858	10	.071	.916	10	.323
	PTZ	.895	14	.095	.931	10	.461	.960	10	.785	.807	14	.006
Up state Index	saline	.895	12	.135	.763	10	.005	.936	10	.511	.869	10	.096
	PTZ	.712	14	.000	.951	10	.683	.888	10	.162	.774	14	.002
Normalized Delta	saline	.783	12	.006	.887	10	.157	.913	10	.302	.848	10	.055
	PTZ	.951	14	.576	.969	10	.877	.829	10	.033	.930	14	.301
Normalized Theta	saline	.897	12	.144	.985	10	.986	.948	10	.647	.934	10	.488
	PTZ	.915	14	.188	.931	10	.458	.927	10	.415	.831	14	.012
Normalized Alpha	saline	.522	12	.000	.972	10	.908	.934	10	.485	.839	10	.043
	PTZ	.916	14	.190	.917	10	.330	.974	10	.928	.929	14	.298
Normalized Beta	saline	.925	12	.329	.797	10	.013	.910	10	.284	.875	10	.113
	PTZ	.904	14	.129	.829	10	.033	.952	10	.695	.958	14	.692
Normalized Gamma	saline	.952	12	.674	.933	10	.481	.849	10	.056	.921	10	.367
	PTZ	.966	14	.820	.897	10	.202	.938	10	.534	.939	14	.411

Table 11. Output (significance) of Shapiro-Wilki's normality tests of distribution of data within each group. Data is color-coded darker colors indicating lower p values.

			S1 cortex				M1 cortex			
		Injections	Mean	SD	Median	IQR	Mean	SD	Median	IQR
Single Injections	Occurrence	saline	0.97	0.58	0.80	1.01	0.58	0.40	0.36	0.74
		PTZ	0.75	0.62	0.52	0.64	1.14	0.56	1.14	1.14
	Duration	saline	1.35	0.23	1.35	0.23	1.50	0.43	1.51	0.70
		PTZ	1.42	0.33	1.44	0.69	1.82	0.58	1.59	0.90
	Amplitude	saline	-64.83	24.78	-65.00	33.00	-49.70	12.31	-51.00	23.25
		PTZ	-54.64	20.68	-51.00	31.50	-64.00	18.18	-62.50	10.25
	RectifiedArea	saline	0.09	0.04	0.08	0.05	0.08	0.04	0.09	0.05
		PTZ	0.08	0.04	0.08	0.06	0.12	0.04	0.13	0.08
	Up state Index	saline	0.07	0.02	0.08	0.04	0.04	0.03	0.03	0.03
		PTZ	0.07	0.08	0.05	0.06	0.14	0.09	0.12	0.15
	NormalizedDelta	saline	0.40	0.14	0.41	0.10	0.38	0.08	0.38	0.08
		PTZ	0.42	0.06	0.41	0.10	0.40	0.06	0.40	0.06
	NormalizedTheta	saline	0.14	0.04	0.14	0.06	0.13	0.04	0.14	0.06
		PTZ	0.14	0.04	0.14	0.06	0.10	0.02	0.11	0.03
	NormalizedAlpha	saline	0.07	0.04	0.06	0.01	0.06	0.02	0.06	0.04
		PTZ	0.06	0.01	0.06	0.02	0.04	0.01	0.04	0.03
	NormalizedBeta	saline	0.09	0.03	0.09	0.04	0.11	0.06	0.12	0.07
		PTZ	0.09	0.03	0.09	0.04	0.08	0.04	0.08	0.02
	NormalizedGamma	saline	0.09	0.05	0.09	0.07	0.09	0.04	0.09	0.06
		PTZ	0.10	0.04	0.10	0.07	0.09	0.04	0.09	0.03
Multiple Injections			S1 cortex				M1 cortex			
		Injections	Mean	SD	Median	IQR	Mean	SD	Median	IQR
	Occurrence	saline	0.74	0.26	0.76	0.40	0.90	0.56	0.87	0.90
		PTZ	0.91	0.56	0.73	0.72	1.06	0.62	0.97	1.36
	Duration	saline	1.29	0.34	1.31	0.62	1.46	0.47	1.52	0.82
		PTZ	1.35	0.32	1.30	0.43	1.63	0.51	1.54	0.84
	Amplitude	saline	-74.50	30.79	-71.50	62.00	-53.50	16.91	-56.00	29.75
		PTZ	-78.80	31.78	-78.00	44.00	-55.07	23.63	-51.00	15.50
	RectifiedArea	saline	0.17	0.07	0.21	0.14	0.11	0.07	0.11	0.09
		PTZ	0.19	0.08	0.20	0.14	0.11	0.07	0.11	0.08
	Up state Index	saline	0.13	0.08	0.13	0.12	0.10	0.09	0.07	0.14
		PTZ	0.18	0.13	0.12	0.20	0.13	0.12	0.07	0.16
	NormalizedDelta	saline	0.45	0.07	0.44	0.13	0.44	0.18	0.45	0.14
		PTZ	0.44	0.07	0.41	0.15	0.48	0.12	0.49	0.12
	NormalizedTheta	saline	0.13	0.05	0.13	0.08	0.10	0.06	0.10	0.06
		PTZ	0.14	0.03	0.15	0.04	0.13	0.05	0.12	0.04
	NormalizedAlpha	saline	0.05	0.02	0.05	0.04	0.04	0.02	0.05	0.03
		PTZ	0.06	0.01	0.06	0.01	0.04	0.02	0.05	0.02
	NormalizedBeta	saline	0.08	0.03	0.08	0.05	0.08	0.04	0.07	0.04
		PTZ	0.08	0.01	0.08	0.02	0.08	0.03	0.08	0.06
	NormalizedGamma	saline	0.08	0.04	0.06	0.07	0.09	0.05	0.08	0.05
		PTZ	0.08	0.03	0.08	0.05	0.07	0.05	0.08	0.09

Table 12. Data of all ten (10) parameters of spontaneous Up states per each experimental group presented as mean and standard deviation (SD) as well as median and interquartile range (IQR). Although mean ± sd and median (IQR) are used to describe mainly normally and non-normally distributed data, respectively, we have included both descriptives for each parameter. The normality of distribution of data for each group can be seen in the preceding table 11.

3.1.2. Three-way ANOVA

3-way ANOVA: number of injections * cortex * injections

Between Subjects Factors		
Factor	Level	N
Injections	Saline	42
	PTZ	48
Cortex	S1	46
	M1	44
Number Of Injections	single	46
	multiple	44

Source	Dependent variable	Sum of Squares	df	Mean Square	F	Sig.
Number of Injections * Cortex * Injections	ARTOccurrence	2074.083	1	2074.083	2.915	.092
	Duration	.028	1	.028	.165	.686
	ARTamplitude	2251.540	1	2251.540	3.166	.079
	ARTRectifiedArea	860.328	1	860.328	1.191	.278
	ART Up state index	2998.295	1	2998.295	4.458	.038
	ARTNormalizedDelta	320.950	1	320.950	.441	.509
	ARTNormalizedTheta	641.627	1	641.627	.880	.351
	ARTNormalizedAlpha	230.086	1	230.086	.324	.571
	ARTNormalizedBeta	128.551	1	128.551	.175	.677
	NormalizedGamma	.000	1	.000	.060	.808
Error	ARTOccurrence	58351.338	82	711.602		
	Duration	14.150	82	.173		
	ARTamplitude	58313.193	82	711.136		
	ARTRectifiedArea	59215.095	82	722.135		
	ART Up state index	55150.681	82	672.569		
	ARTNormalizedDelta	59734.095	82	728.465		
	ARTNormalizedTheta	59776.786	82	728.985		
	ARTNormalizedAlpha	58304.674	82	711.033		
	ARTNormalizedBeta	60274.681	82	735.057		
	NormalizedGamma	.150	82	.002		

Table 13. The significance of interaction between the effects of "number of injection", "type of cortex" and "injections" for all ten (10) parameters of spontaneous Up states. Data that were not normally distributed (Table 11) were previously transformed according to the Aligned Rank Transformation (ART) rules.

3.2. Simple effects analysis

3.2.1. Simple effects of seizures

3.2.1.1. Simple effect of seizures on Up state Index

Estimated Marginal Means

Number Of Injections * Cortex * Injections

Estimates

Dependent Variable:ARTUpstateIndexNumberXCortexBYInjections

NumberOfInjections	Cortex	Injections	Mean	Std. Error	95% Confidence Interval	
					Lower Bound	Upper Bound
single	S1	saline	16.583	1.851	12.901	20.265
		PTZ	10.857	1.714	7.448	14.266
	M1	saline	6.700	2.028	2.666	10.734
		PTZ	14.300	2.028	10.266	18.334
multiple	S1	saline	10.000	2.028	5.966	14.034
		PTZ	11.000	2.028	6.966	15.034
	M1	saline	11.300	2.028	7.266	15.334
		PTZ	13.357	1.714	9.948	16.766

Pairwise Comparisons

Dependent Variable: ARTUpstateIndexNumberXCortexBYInjections

NumberOfInjections	Cortex	(I) Injections	(J) Injections	Mean Difference (I-J)	Std. Error	Sig.[a]	95% Confidence Interval for Difference[a]	
							Lower Bound	Upper Bound
single	S1	saline	PTZ	5.726*	2.522	.026	.708	10.744
		PTZ	saline	-5.726*	2.522	.026	-10.744	-.708
	M1	saline	PTZ	-7.600*	2.867	.010	-13.304	-1.896
		PTZ	saline	7.600*	2.867	.010	1.896	13.304
multiple	S1	saline	PTZ	-1.000	2.867	.728	-6.704	4.704
		PTZ	saline	1.000	2.867	.728	-4.704	6.704
	M1	saline	PTZ	-2.057	2.655	.441	-7.338	3.224
		PTZ	saline	2.057	2.655	.441	-3.224	7.338

Based on estimated marginal means

*. The mean difference is significant at the .05 level.

a. Adjustment for multiple comparisons: Bonferroni.

Univariate Tests

Dependent Variable: ARTUpstateIndexNumberXCortexBYInjections

NumberOfInjections	Cortex		Sum of Squares	df	Mean Square	F	Sig.
single	S1	Contrast	211.869	1	211.869	5.154	**.026**
		Error	3371.145	82	41.112		
	M1	Contrast	288.800	1	288.800	7.025	**.010**
		Error	3371.145	82	41.112		
multiple	S1	Contrast	5.000	1	5.000	.122	.728
		Error	3371.145	82	41.112		
	M1	Contrast	24.686	1	24.686	.600	.441
		Error	3371.145	82	41.112		

Each F tests the simple effects of Injections within each level combination of the other effects shown. These tests are based on the linearly independent pairwise comparisons among the estimated marginal means.

3.3. *Analysis of two-way interaction: number of injections * injections*

3.3.1. *Normality of data distribution and descriptive of data for each group (number of injections * injections)*

		Single Injections			Multiple Injections		
		Shapiro-Wilk			Shapiro-Wilk		
	Injections	Statistic	df	Sig.	Statistic	df	Sig.
Occurrence	saline	.888	22	.017	.945	20	.303
	PTZ	.903	24	.025	.890	24	.013
Duration	saline	.967	22	.650	.961	20	.561
	PTZ	.921	24	.062	.935	24	.125
Amplitude	saline	.889	22	.018	.933	20	.174
	PTZ	.891	24	.014	.924	24	.072
RectifiedArea	saline	.958	22	.449	.926	20	.128
	PTZ	.939	24	.152	.918	24	.054
NormalizedDelta	saline	.818	22	.001	.859	20	.007
	PTZ	.968	24	.620	.934	24	.122
NormalizedTheta	saline	.963	22	.547	.966	20	.678
	PTZ	.929	24	.093	.921	24	.061
NormalizedAlpha	saline	.735	22	.000	.970	20	.763
	PTZ	.951	24	.290	.918	24	.053
NormalizedBeta	saline	.856	22	.004	.929	20	.145
	PTZ	.901	24	.023	.981	24	.917
NormalizedGamma	saline	.962	22	.541	.952	20	.403
	PTZ	.969	24	.640	.965	24	.548

Table 14. Output (significance) of Shapiro-Wilki's normality tests of distribution of data within each group. Data is color-coded darker colors indicating lower p values.

		Single				Multiple			
	Injections	Mean	SD	Median	IQR	Mean	SD	Median	IQR
Occurrence	saline	0.79	0.53	0.66	0.74	1.38	0.41	1.40	0.74
	PTZ	0.91	0.61	0.73	0.83	1.51	0.46	1.40	0.71
Duration	saline	1.42	0.34	1.36	0.51	-64.00	26.47	-60.00	33.50
	PTZ	1.59	0.49	1.54	0.59	-64.96	29.21	-56.50	40.00
Amplitude	saline	-57.95	21.12	-55.00	25.25	0.14	0.08	0.13	0.14
	PTZ	-58.54	19.83	-59.00	23.25	0.15	0.08	0.13	0.13
RectifiedArea	saline	0.09	0.04	0.09	0.05	0.44	0.13	0.45	0.11
	PTZ	0.10	0.04	0.09	0.07	0.46	0.10	0.45	0.13
Normalized Delta	saline	0.39	0.12	0.38	0.08	0.11	0.06	0.11	0.06
	PTZ	0.41	0.06	0.41	0.08	0.13	0.04	0.14	0.04
Normalized Theta	saline	0.14	0.04	0.14	0.06	0.04	0.02	0.05	0.03
	PTZ	0.12	0.04	0.12	0.04	0.05	0.02	0.05	0.02
Normalized Alpha	saline	0.06	0.03	0.06	0.02	0.08	0.03	0.07	0.05
	PTZ	0.05	0.02	0.05	0.02	0.08	0.03	0.08	0.03
Normalized Beta	saline	0.10	0.04	0.10	0.06	0.08	0.04	0.07	0.06
	PTZ	0.09	0.03	0.09	0.03	0.07	0.04	0.08	0.06
Normalized Gamma	saline	0.09	0.04	0.09	0.06	0.00	0.00	0.00	0.00
	PTZ	0.09	0.04	0.09	0.04	0.00	0.00	0.00	0.00

Table 15. Data of all ten (10) parameters of spontaneous Up states per each experimental group presented as mean ± standard deviation (sd) and median with interquartile range (IQR). Although mean ± sd and median (IQR) are used to describe mainly normally and non-normally distributed data, respectively, we have included both descriptive for each parameter. The normality of distribution of data for each group can be seen in preceding table 14.

3.3.2. Two-way ANOVA

2-way ANOVA: number of injections * injections

Between Subjects Factors		
Factor	Level	N
Injections	Saline	42
	PTZ	48
Number Of Injections	single	46
	multiple	44

Source	Dependent variable	Sum of Squares	df	Mean Square	F	Sig.
Number Of Injections * Injections	ARTOccurrenceNumberXInjections	.272	1	.272	.000	.984
	Duration	.008	1	.008	.043	.837
	ARTAmplitudeNumberXInjections	3.821	1	3.821	.005	.941
	RectifiedArea	.000	1	.000	.035	.851
	ARTNormalizedDeltaNumberXInjections	110.835	1	110.835	.159	.691
	NormalizedTheta	.006	1	.006	2.959	.089
	ARTNormalizedAlphaNumberXinjections	1463.358	1	1463.358	2.165	.145
	ARTNormalizedBetaNumberXInjections	799.536	1	799.536	1.152	.286
	NormalizedGamma	.000	1	.000	.146	.704
Error	ARTOccurrenceNumberXInjections	60503.946	86	703.534		
	Duration	15.812	86	.184		
	ARTAmplitudeNumberXInjections	60472.431	86	703.168		
	RectifiedArea	.335	86	.004		
	ARTNormalizedDeltaNumberXInjections	59879.556	86	696.274		
	NormalizedTheta	.173	86	.002		
	ARTNormalizedAlphaNumberXinjections	58140.992	86	676.058		
	ARTNormalizedBetaNumberXInjections	59694.080	86	694.117		
	NormalizedGamma	.151	86	.002		

Table 16. The significance of interaction between the effects of "number of injection" and "injections" for nine parameters of spontaneous Up states. Data that were not normally distributed (Table 14) were previously transformed according to the Aligned Rank Transformation (ART) rules.

4. Younger ages are more resilient to the long-term effects of ELS on local cortical network activity

4.1. Analysis of three-way interaction: age of injections * cortex * injections

4.1.1. Normality tests of data distribution and descriptives of data for each group (age of injections * cortex * injections)

	Age Of Injections	P9-15						P19-23					
	Cortex	S1			M1			S1			M1		
		Shapiro-Wilk			Shapiro-Wilk			Shapiro-Wilk			Shapiro-Wilk		
	Injections	Statistic	df	Sig.	Statistic	df	Sig.	Statistic	df	Sig.	Statistic	df	Sig.
Occurrence	saline	.984	10	.982	.967	10	.866	.870	13	.052	.867	6	.214
	PTZ	.822	10	.027	.887	14	.073	.959	11	.759	.667	5	.004
Duration	saline	.959	10	.771	.936	10	.511	.854	13	.032	.938	6	.642
	PTZ	.942	10	.578	.953	14	.607	.926	11	.372	.940	5	.669
Amplitude	saline	.915	10	.316	.946	10	.623	.839	13	.020	.866	6	.211
	PTZ	.975	10	.931	.834	14	.014	.939	11	.513	.938	5	.655
Rectified Area	saline	.858	10	.071	.916	10	.323	.742	13	.002	.915	6	.470
	PTZ	.960	10	.785	.807	14	.006	.779	11	.005	.855	5	.211
Up state Index	saline	.936	10	.511	.869	10	.096	.459	13	.000	.920	6	.504
	PTZ	.888	10	.162	.774	14	.002	.716	11	.001	.788	5	.065
Normalized Delta	saline	.913	10	.302	.848	10	.055	.917	13	.226	.885	6	.294
	PTZ	.829	10	.033	.930	14	.301	.939	11	.513	.996	5	.997
Normalized Theta	saline	.948	10	.647	.934	10	.488	.951	13	.619	.884	6	.289
	PTZ	.927	10	.415	.831	14	.012	.966	11	.843	.909	5	.462
Normalized Alpha	saline	.934	10	.485	.839	10	.043	.935	13	.393	.859	6	.186
	PTZ	.974	10	.928	.929	14	.298	.952	11	.665	.923	5	.548
Normalized Beta	saline	.910	10	.284	.875	10	.113	.936	13	.412	.940	6	.661
	PTZ	.952	10	.695	.958	14	.692	.954	11	.697	.995	5	.994
Normalized Gamma	saline	.849	10	.056	.921	10	.367	.922	13	.264	.920	6	.505
	PTZ	.938	10	.534	.939	14	.411	.956	11	.723	.727	5	.018

Table 17. Output (significance) of Shapiro-Wilki's normality tests of distribution of data within each group. Data is color-coded darker colors indicating lower p values.

			S1 cortex				M1 cortex			
		Injections	Mean	SD	Median	IQR	Mean	SD	Median	IQR
P9-15	Occurrence	saline	0.74	0.26	0.76	0.40	0.90	0.56	0.87	0.90
		PTZ	0.91	0.56	0.73	0.72	1.06	0.62	0.97	1.36
	Duration	saline	1.29	0.34	1.31	0.62	1.46	0.47	1.52	0.82
		PTZ	1.35	0.32	1.30	0.43	1.63	0.51	1.54	0.84
	Amplitude	saline	-74.50	30.79	-71.50	62.00	-53.50	16.91	-56.00	29.75
		PTZ	-78.80	31.78	-78.00	44.00	-55.07	23.63	-51.00	15.50
	RectifiedArea	saline	0.17	0.07	0.21	0.14	0.11	0.07	0.11	0.09
		PTZ	0.19	0.08	0.20	0.14	0.11	0.07	0.11	0.08
	Up state Index	saline	0.13	0.08	0.13	0.12	0.10	0.09	0.07	0.14
		PTZ	0.18	0.13	0.12	0.20	0.13	0.12	0.07	0.16
	NormalizedDelta	saline	0.45	0.07	0.44	0.13	0.44	0.18	0.45	0.14
		PTZ	0.44	0.07	0.41	0.15	0.48	0.12	0.49	0.12
	NormalizedTheta	saline	0.13	0.05	0.13	0.08	0.10	0.06	0.10	0.06
		PTZ	0.14	0.03	0.15	0.04	0.13	0.05	0.12	0.04
	NormalizedAlpha	saline	0.05	0.02	0.05	0.04	0.04	0.02	0.05	0.03
		PTZ	0.06	0.01	0.06	0.01	0.04	0.02	0.05	0.02
	NormalizedBeta	saline	0.08	0.03	0.08	0.05	0.08	0.04	0.07	0.04
		PTZ	0.08	0.01	0.08	0.02	0.08	0.03	0.08	0.06
	NormalizedGamma	saline	0.08	0.04	0.06	0.07	0.09	0.05	0.08	0.05
		PTZ	0.08	0.03	0.08	0.05	0.07	0.05	0.08	0.09
P19-23			S1 cortex				M1 cortex			
		Injections	Mean	SD	Median	IQR	Mean	SD	Median	IQR
	Occurrence	saline	1.36	0.71	1.21	0.87	0.55	0.39	0.48	0.46
		PTZ	1.04	0.45	1.04	0.59	0.48	0.45	0.34	0.59
	Duration	saline	1.61	0.32	1.53	0.39	1.45	0.62	1.32	0.82
		PTZ	1.31	0.35	1.21	0.62	1.56	0.53	1.42	0.92
	Amplitude	saline	-60.46	27.78	-54.00	28.00	-68.67	25.34	-78.50	48.25
		PTZ	-73.64	31.83	-64.00	61.00	-52.40	19.33	-50.00	32.00
	RectifiedArea	saline	0.14	0.08	0.12	0.06	0.10	0.05	0.12	0.10
		PTZ	0.15	0.12	0.12	0.09	0.11	0.06	0.11	0.10
	Up state Index	saline	0.22	0.30	0.11	0.09	0.06	0.06	0.06	0.08
		PTZ	0.18	0.21	0.14	0.12	0.05	0.05	0.04	0.08
	NormalizedDelta	saline	0.53	0.07	0.52	0.11	0.56	0.08	0.54	0.16
		PTZ	0.53	0.08	0.54	0.13	0.57	0.11	0.57	0.21
	NormalizedTheta	saline	0.16	0.02	0.16	0.02	0.18	0.05	0.17	0.10
		PTZ	0.21	0.04	0.21	0.06	0.15	0.02	0.15	0.03
	NormalizedAlpha	saline	0.07	0.01	0.06	0.02	0.06	0.02	0.06	0.03
		PTZ	0.07	0.02	0.07	0.03	0.06	0.03	0.06	0.04
	NormalizedBeta	saline	0.13	0.04	0.12	0.07	0.10	0.04	0.10	0.06
		PTZ	0.10	0.03	0.10	0.05	0.10	0.05	0.11	0.08
	NormalizedGamma	saline	0.12	0.04	0.11	0.08	0.09	0.06	0.08	0.11
		PTZ	0.09	0.03	0.09	0.04	0.12	0.06	0.11	0.08

Table 18. Data of all ten (10) parameters of spontaneous Up states per each experimental group presented as mean ± standard deviation (sd) and median with interquartile range (IQR). Although mean ± sd and median (IQR) are used to describe mainly normally and non-normally distributed data, respectively, we have included both descriptive for each parameter. The normality of distribution of data for each group can be seen in preceding table 17.

4.1.2. Three-way ANOVA

3-way ANOVA: age * cortex * injections

Between Subjects Factors

Factor	Level	N
Injections	Saline	39
	PTZ	40
Cortex	S1	44
	M1	35
Age Of Injections	P9-15	44
	P19-23	35

Source	Dependent variable	Sum of Squares	df	Mean Square	F	Sig.
Number of Injections * Cortex * Injections	ARTOccurrenceAgeXCortexXInjections	2.503	1	2.503	.004	.947
	ARTDurationAgeXCortexXInjections	235.936	1	235.936	.413	.522
	ARTAmplitudeAgeXCortexXInjections	531.494	1	531.494	.939	.336
	ARTRectifiedAreaAgeXCortexXInjections	17.931	1	17.931	.032	.859
	ART Up state IndexAgeXCortexXInjections	42.329	1	42.329	.083	.774
	ARTDeltaAgeXCortexXInjections	129.357	1	129.357	.225	.637
	ARTNormalizedThetaAgeXCortexXInjections	2485.449	1	2485.449	4.620	.035
	ARTThetaAgeXCortexXInjections	233.966	1	233.966	.409	.524
	NormalizedBeta	.001	1	.001	.778	.381
	ARTGammaAgeXCortexXInjections	2279.548	1	2279.548	4.184	.045
Error	ARTOccurrenceAgeXCortexXInjections	40299.159	71	567.594		
	ARTDurationAgeXCortexXInjections	40555.710	71	571.207		
	ARTAmplitudeAgeXCortexXInjections	40193.421	71	566.105		
	ARTRectifiedAreaAgeXCortexXInjections	39973.312	71	563.004		
	ART Up state IndexAgeXCortexXInjections	36271.265	71	510.863		
	ARTDeltaAgeXCortexXInjections	40803.276	71	574.694		
	ARTNormalizedThetaAgeXCortexXInjections	38199.367	71	538.019		
	ARTThetaAgeXCortexXInjections	40570.685	71	571.418		
	NormalizedBeta	.077	71	.001		
	ARTGammaAgeXCortexXInjections	38677.995	71	544.760		

Table 19. The significance of interaction between the effects of "age of injection", "type of cortex" and "injections" for all ten (10) parameters of spontaneous Up states. Data that were not normally distributed (Table 17) were previously transformed according to the Aligned Rank Transformation (ART) rules.

4.2. Simple effect analysis

4.2.1. Simple effects of seizures

4.2.1.1. Simple effects of seizures on Theta

Estimated Marginal Means

Injections * AgeOfInjections * Cortex

Estimates

Dependent Variable:ARTThetaAgeXCortexBYInjections

Injections	AgeOfInjections	Cortex	Mean	Std. Error	95% Confidence Interval Lower Bound	95% Confidence Interval Upper Bound
saline	P9-15	S1	9.050	1.839	5.383	12.717
		M1	10.200	1.839	6.533	13.867
	P19-23	S1	8.385	1.613	5.169	11.600
		M1	7.083	2.374	2.350	11.817
PTZ	P9-15	S1	11.950	1.839	8.283	15.617
		M1	14.143	1.554	11.044	17.242
	P19-23	S1	17.364	1.753	13.868	20.860
		M1	4.700	2.601	-.485	9.885

Pairwise Comparisons

Dependent Variable:ARTThetaAgeXCortexBYInjections

AgeOfInjections	Cortex	(I) Injections	(J) Injections	Mean Difference (I-J)	Std. Error	Sig.[a]	95% Confidence Interval for Difference[a] Lower Bound	95% Confidence Interval for Difference[a] Upper Bound
P9-15	S1	saline	PTZ	-2.900	2.601	.269	-8.085	2.285
		PTZ	saline	2.900	2.601	.269	-2.285	8.085
	M1	saline	PTZ	-3.943	2.408	.106	-8.744	.858
		PTZ	saline	3.943	2.408	.106	-.858	8.744
P19-23	S1	saline	PTZ	-8.979*	2.382	.000	-13.729	-4.229
		PTZ	saline	8.979*	2.382	.000	4.229	13.729
	M1	saline	PTZ	2.383	3.521	.501	-4.638	9.404
		PTZ	saline	-2.383	3.521	.501	-9.404	4.638

Based on estimated marginal means

a. Adjustment for multiple comparisons: Bonferroni.

*. The mean difference is significant at the .05 level.

Univariate Tests

Dependent Variable:ARTThetaAgeXCortexBYInjections

AgeOfInjections	Cortex		Sum of Squares	df	Mean Square	F	Sig.
P9-15	S1	Contrast	42.050	1	42.050	1.244	.269
		Error	2400.895	71	33.815		
	M1	Contrast	90.686	1	90.686	2.682	.106
		Error	2400.895	71	33.815		
P19-23	S1	Contrast	480.378	1	480.378	14.206	**.000**
		Error	2400.895	71	33.815		
	M1	Contrast	15.492	1	15.492	.458	.501
		Error	2400.895	71	33.815		

Each F tests the simple effects of Injections within each level combination of the other effects shown. These tests are based on the linearly independent pairwise comparisons among the estimated marginal means.

4.2.1.2. Simple effect of seizures on Gamma

Estimated Marginal Means

Injections * AgeOfInjections * Cortex

Estimates

Dependent Variable:ARTGammaAgeXCortexBYInjections

Injections	AgeOfInjections	Cortex	Mean	Std. Error	95% Confidence Interval	
					Lower Bound	Upper Bound
saline	P9-15	S1	10.100	1.991	6.129	14.071
		M1	13.350	1.991	9.379	17.321
	P19-23	S1	15.077	1.747	11.594	18.559
		M1	4.917	2.571	-.209	10.043
PTZ	P9-15	S1	10.900	1.991	6.929	14.871
		M1	11.893	1.683	8.537	15.249
	P19-23	S1	9.455	1.899	5.669	13.240
		M1	7.300	2.816	1.685	12.915

Pairwise Comparisons

Dependent Variable:ARTGammaAgeXCortexBYInjections

AgeOfInjections	Cortex	(I) Injections	(J) Injections	Mean Difference (I-J)	Std. Error	Sig.[a]	95% Confidence Interval for Difference[a]	
							Lower Bound	Upper Bound
P9-15	S1	saline	PTZ	-.800	2.816	.777	-6.415	4.815
		PTZ	saline	.800	2.816	.777	-4.815	6.415
	M1	saline	PTZ	1.457	2.607	.578	-3.742	6.656
		PTZ	saline	-1.457	2.607	.578	-6.656	3.742
P19-23	S1	saline	PTZ	5.622*	2.580	.033	.478	10.766
		PTZ	saline	-5.622*	2.580	.033	-10.766	-.478
	M1	saline	PTZ	-2.383	3.813	.534	-9.987	5.220
		PTZ	saline	2.383	3.813	.534	-5.220	9.987

Based on estimated marginal means

a. Adjustment for multiple comparisons: Bonferroni.

*. The mean difference is significant at the .05 level.

Univariate Tests

Dependent Variable:ARTGammaAgeXCortexBYInjections

AgeOfInjections	Cortex		Sum of Squares	df	Mean Square	F	Sig.
P9-15	S1	Contrast	3.200	1	3.200	.081	.777
		Error	2815.573	71	39.656		
	M1	Contrast	12.386	1	12.386	.312	.578
		Error	2815.573	71	39.656		
P19-23	S1	Contrast	188.350	1	188.350	4.750	**.033**
		Error	2815.573	71	39.656		
	M1	Contrast	15.492	1	15.492	.391	.534
		Error	2815.573	71	39.656		

Each F tests the simple effects of Injections within each level combination of the other effects shown. These tests are based on the linearly independent pairwise comparisons among the estimated marginal means.

4.2.2. Simple effect of age of seizures ("age of injections")

4.2.2.1. Simple effect of age of seizures on Theta

Estimated Marginal Means

Injections * Age Of Injections * Cortex

Estimates

Dependent Variable:ARTThetaCortexXInjectionsBYage

Injections	AgeOfInjections	Cortex	Mean	Std. Error	95% Confidence Interval	
					Lower Bound	Upper Bound
saline	P9-15	S1	8.950	1.633	5.695	12.205
		M1	6.050	1.633	2.795	9.305
	P19-23	S1	14.346	1.432	11.491	17.201
		M1	12.583	2.108	8.381	16.786
PTZ	P9-15	S1	6.750	1.633	3.495	10.005
		M1	8.607	1.380	5.856	11.358
	P19-23	S1	14.864	1.557	11.760	17.967
		M1	13.900	2.309	9.296	18.504

Pairwise Comparisons

Dependent Variable:ARTThetaCortexXInjectionsBYage

Injections	Cortex	(I) AgeOfInjections	(J) AgeOfInjections	Mean Difference (I-J)	Std. Error	Sig.[a]	95% Confidence Interval for Difference[a]	
							Lower Bound	Upper Bound
saline	S1	P9-15	P19-23	-5.396*	2.171	.015	-9.726	-1.066
		P19-23	P9-15	5.396*	2.171	.015	1.066	9.726
	M1	P9-15	P19-23	-6.533*	2.666	.017	-11.849	-1.218
		P19-23	P9-15	6.533*	2.666	.017	1.218	11.849
PTZ	S1	P9-15	P19-23	-8.114*	2.256	.001	-12.611	-3.616
		P19-23	P9-15	8.114*	2.256	.001	3.616	12.611
	M1	P9-15	P19-23	-5.293	2.690	.053	-10.656	.070
		P19-23	P9-15	5.293	2.690	.053	-.070	10.656

Based on estimated marginal means

*. The mean difference is significant at the .05 level.

a. Adjustment for multiple comparisons: Bonferroni.

Univariate Tests

Dependent Variable:ARTThetaCortexXInjectionsBYage

Injections	Cortex		Sum of Squares	df	Mean Square	F	Sig.
saline	S1	Contrast	164.583	1	164.583	6.175	.015
		Error	1892.310	71	26.652		
	M1	Contrast	160.067	1	160.067	6.006	.017
		Error	1892.310	71	26.652		
PTZ	S1	Contrast	344.830	1	344.830	12.938	.001
		Error	1892.310	71	26.652		
	M1	Contrast	103.211	1	103.211	3.872	.053
		Error	1892.310	71	26.652		

Each F tests the simple effects of AgeOfInjections within each level combination of the other effects shown. These tests are based on the linearly independent pairwise comparisons among the estimated marginal means.

4.2.2.2.Simple effect of age of seizures on Gamma

Estimated Marginal Means

Injections * AgeOfInjections * Cortex

Estimates

Dependent Variable:ARTGammaCortexXInjectionsBYAge

Injections	AgeOfInjections	Cortex	Mean	Std. Error	95% Confidence Interval	
					Lower Bound	Upper Bound
saline	P9-15	S1	8.250	1.811	4.640	11.860
		M1	8.600	1.811	4.990	12.210
	P19-23	S1	14.885	1.588	11.718	18.051
		M1	8.333	2.338	3.672	12.994
PTZ	P9-15	S1	10.200	1.811	6.590	13.810
		M1	8.857	1.530	5.806	11.908
	P19-23	S1	11.727	1.726	8.285	15.170
		M1	13.200	2.561	8.094	18.306

Pairwise Comparisons

Dependent Variable:ARTGammaCortexXInjectionsBYAge

Injections	Cortex	(I) AgeOfInjections	(J) AgeOfInjections	Mean Difference (I-J)	Std. Error	Sig.[a]	95% Confidence Interval for Difference[a] Lower Bound	Upper Bound
saline	S1	P9-15	P19-23	-6.635*	2.408	.007	-11.437	-1.832
		P19-23	P9-15	6.635*	2.408	.007	1.832	11.437
	M1	P9-15	P19-23	.267	2.957	.928	-5.629	6.162
		P19-23	P9-15	-.267	2.957	.928	-6.162	5.629
PTZ	S1	P9-15	P19-23	-1.527	2.502	.543	-6.516	3.461
		P19-23	P9-15	1.527	2.502	.543	-3.461	6.516
	M1	P9-15	P19-23	-4.343	2.983	.150	-10.291	1.605
		P19-23	P9-15	4.343	2.983	.150	-1.605	10.291

Based on estimated marginal means

*. The mean difference is significant at the .05 level.

a. Adjustment for multiple comparisons: Bonferroni.

Univariate Tests

Dependent Variable:ARTGammaCortexXInjectionsBYAge

Injections	Cortex		Sum of Squares	df	Mean Square	F	Sig.
saline	S1	Contrast	248.798	1	248.798	7.589	**.007**
		Error	2327.731	71	32.785		
	M1	Contrast	.267	1	.267	.008	.928
		Error	2327.731	71	32.785		
PTZ	S1	Contrast	12.218	1	12.218	.373	.543
		Error	2327.731	71	32.785		
	M1	Contrast	69.486	1	69.486	2.119	.150
		Error	2327.731	71	32.785		

Each F tests the simple effects of AgeOfInjections within each level combination of the other effects shown. These tests are based on the linearly independent pairwise comparisons among the estimated marginal means.

*4.3. Analysis of two-way interaction: age of injections * injections*

*4.3.1. Normality of data distribution and descriptive of data for each group (age of injections * injections)*

Age Of Injections		P10-15			P20-25		
		Shapiro-Wilk			Shapiro-Wilk		
	Injections	Statistic	df	Sig.	Statistic	df	Sig.
Occurrence	saline	.945	20	.303	.893	19	.037
	PTZ	.890	24	.013	.924	16	.197
Duration	saline	.961	20	.561	.917	19	.102
	PTZ	.935	24	.125	.947	16	.441
Amplitude	saline	.933	20	.174	.917	19	.100
	PTZ	.924	24	.072	.921	16	.173
RectifiedArea	saline	.926	20	.128	.790	19	.001
	PTZ	.918	24	.054	.781	16	.002
NetworkIndex	saline	.923	20	.115	.457	19	.000
	PTZ	.833	24	.001	.656	16	.000
NormalizedDelta	saline	.859	20	.007	.959	19	.556
	PTZ	.934	24	.122	.956	16	.593
NormalizedAlpha	saline	.970	20	.763	.923	19	.128
	PTZ	.918	24	.053	.973	16	.883
NormalizedBeta	saline	.929	20	.145	.948	19	.361
	PTZ	.981	24	.917	.982	16	.976

Table 20. Output (significance) of Shapiro-Wilki's normality tests of distribution of data within each group. Data is color-coded darker colors indicating lower p values.

	Age of Injections	P9-15				P19-23			
	Injections	Mean	SD	Median	IQR	Mean	SD	Median	IQR
Occurrence	saline	0.82	0.43	0.82	0.55	1.10	0.73	1.12	0.83
	PTZ	1.00	0.59	0.81	0.86	0.87	0.51	1.01	0.89
Duration	saline	1.38	0.41	1.40	0.74	1.56	0.43	1.45	0.40
	PTZ	1.51	0.46	1.40	0.71	1.39	0.41	1.33	0.62
Amplitude	saline	-64.00	26.47	-60.00	33.50	-63.05	26.61	-60.00	36.00
	PTZ	-64.96	29.21	-56.50	40.00	-67.00	29.64	-61.00	38.00
RectifiedArea	saline	0.14	0.08	0.13	0.14	0.13	0.07	0.12	0.06
	PTZ	0.15	0.08	0.13	0.13	0.13	0.11	0.11	0.09
NetworkIndex	saline	0.12	0.08	0.11	0.13	0.17	0.26	0.10	0.10
	PTZ	0.15	0.12	0.10	0.19	0.14	0.18	0.08	0.11
NormalizedDelta	saline	0.44	0.13	0.45	0.11	0.54	0.08	0.52	0.12
	PTZ	0.46	0.10	0.45	0.13	0.54	0.09	0.55	0.14
NormalizedAlpha	saline	0.04	0.02	0.05	0.03	0.06	0.01	0.06	0.02
	PTZ	0.05	0.02	0.05	0.02	0.07	0.02	0.07	0.03
NormalizedBeta	saline	0.08	0.03	0.07	0.05	0.12	0.04	0.12	0.07
	PTZ	0.08	0.03	0.08	0.03	0.10	0.03	0.10	0.05

Table 21. Data of all ten (10) parameters of spontaneous Up states per each experimental group presented as mean ± standard deviation (sd) and median with interquartile range (IQR). Although mean ± sd and median (IQR) are used to describe mainly normally and non-normally distributed data, respectively, we have included both descriptive for each parameter. The normality of distribution of data for each group can be seen in preceding table 20.

4.3.2. Two-way ANOVA

2-way ANOVA: age of injections * injections

Between Subjects Factors

Factor	Level	N
Injections	Saline	39
	PTZ	40
Age Of Injections	P9-15	44
	P19-23	35

Source	Dependent variable	Sum of Squares	df	Mean Square	F	Sig.
Number Of Injections * Injections	ARTOccurrenceAgeXInjections	844.914	1	844.914	1.577	.213
	Duration	.465	1	.465	2.526	.116
	Amplitude	43.203	1	43.203	.055	.815
	ARTRectifiedAreaAgeXInjections	66.396	1	66.396	.122	.728
	ARTNetworkIndexAgeXInjections	475.832	1	475.832	.912	.343
	ARTDeltaAgeXInjections	88.148	1	88.148	.162	.689
	NormalizedAlpha	.000	1	.000	.000	.986
	NormalizedBeta	.002	1	.002	2.153	.146
	ARTOccurrenceAgeXInjections	844.914	1	844.914	1.577	.213
Error	ARTOccurrenceAgeXInjections	40190.471	75	535.873		
	Duration	13.820	75	.184		
	Amplitude	58863.906	75	784.852		
	ARTRectifiedAreaAgeXInjections	40752.958	75	543.373		
	ARTNetworkIndexAgeXInjections	39122.839	75	521.638		
	ARTDeltaAgeXInjections	40836.933	75	544.492		
	NormalizedAlpha	.025	75	.000		
	NormalizedBeta	.079	75	.001		
	ARTOccurrenceAgeXInjections	40190.471	75	535.873		

Table 22. The significance of interaction between the effects of "age of injection" and "injections" for nine parameters of spontaneous Up states. Data that were not normally distributed (Table 20) were previously transformed according to the Aligned Rank Transformation (ART) rules.

5. The long-term effects of ELS on cortical dynamics depend on their frequency

5.1. Analysis of three-way interaction: number of injections * cortex * injections

5.1.1. Normality tests of data distribution and descriptives of data for each group (number of injections * cortex * injections)

	Cortex	S1						M1					
	Number of Injections	Single			Multiple			Single			Multiple		
		Shapiro-Wilk			Shapiro-Wilk			Shapiro-Wilk			Shapiro-Wilk		
	Injections	Statistic	df	Sig.	Statistic	df	Sig.	Statistic	df	Sig.	Statistic	df	Sig.
Occurrence	saline	.935	9	.535	.870	13	.052	.850	11	.043	.867	6	.214
	PTZ	.971	10	.897	.959	11	.759	.874	9	.136	.667	5	.004
Duration	saline	.965	9	.850	.854	13	.032	.731	11	.001	.938	6	.642
	PTZ	.744	10	.003	.926	11	.372	.907	9	.293	.940	5	.669
Amplitude	saline	.597	9	.000	.839	13	.020	.775	11	.004	.866	6	.211
	PTZ	.792	10	.012	.939	11	.513	.896	9	.229	.938	5	.655
Rectified Area	saline	.760	9	.007	.742	13	.002	.786	11	.006	.915	6	.470
	PTZ	.952	10	.688	.779	11	.005	.905	9	.285	.855	5	.211
Up state Index	saline	.855	9	.085	.459	13	.000	.654	11	.000	.920	6	.504
	PTZ	.875	10	.115	.716	11	.001	.945	9	.632	.788	5	.065
Normalized Delta	saline	.944	9	.625	.917	13	.226	.935	11	.469	.885	6	.294
	PTZ	.937	10	.525	.939	11	.513	.971	9	.899	.996	5	.997
Normalized Theta	saline	.926	9	.444	.951	13	.619	.863	11	.063	.884	6	.289
	PTZ	.950	10	.669	.966	11	.843	.949	9	.680	.909	5	.462
Normalized Alpha	saline	.841	9	.059	.935	13	.393	.907	11	.226	.859	6	.186
	PTZ	.927	10	.420	.952	11	.665	.864	9	.107	.923	5	.548
Normalized Beta	saline	.904	9	.276	.936	13	.412	.975	11	.934	.940	6	.661
	PTZ	.975	10	.931	.954	11	.697	.910	9	.314	.995	5	.994
Normalized Gamma	saline	.796	9	.018	.922	13	.264	.948	11	.624	.920	6	.505
	PTZ	.826	10	.030	.956	11	.723	.957	9	.766	.727	5	.018

Table 23. Output (significance) of Shapiro-Wilki's normality tests of distribution of data within each group. Data is color-coded darker colors indicating lower p values.

			Single				Multiple			
		Injections	Mean	SD	Median	IQR	Mean	SD	Median	IQR
S1 cortex	Occurrence	saline	1.35	0.84	1.32	1.53	1.36	0.71	1.21	0.87
		PTZ	1.44	0.77	1.41	1.41	1.04	0.45	1.04	0.59
	Duration	saline	1.52	0.36	1.47	0.45	1.61	0.32	1.53	0.39
		PTZ	1.48	0.31	1.52	0.21	1.31	0.35	1.21	0.62
	Amplitude	saline	-86.89	86.25	-51.00	39.50	-60.46	27.78	-54.00	28.00
		PTZ	-68.10	30.10	-54.00	40.50	-73.64	31.83	-64.00	61.00
	RectifiedArea	saline	0.21	0.13	0.18	0.12	0.14	0.08	0.12	0.06
		PTZ	0.18	0.07	0.17	0.08	0.15	0.12	0.12	0.09
	Up state Index	saline	0.32	0.30	0.18	0.49	0.22	0.30	0.11	0.09
		PTZ	0.30	0.24	0.24	0.27	0.18	0.21	0.14	0.12
	NormalizedDelta	saline	0.57	0.13	0.61	0.19	0.53	0.07	0.52	0.11
		PTZ	0.58	0.12	0.61	0.18	0.53	0.08	0.54	0.13
	NormalizedTheta	saline	0.17	0.03	0.16	0.04	0.16	0.02	0.16	0.02
		PTZ	0.15	0.03	0.15	0.06	0.21	0.04	0.21	0.06
	NormalizedAlpha	saline	0.07	0.02	0.07	0.02	0.07	0.01	0.06	0.02
		PTZ	0.06	0.02	0.05	0.03	0.07	0.02	0.07	0.03
	NormalizedBeta	saline	0.10	0.06	0.08	0.07	0.13	0.04	0.12	0.07
		PTZ	0.10	0.04	0.09	0.06	0.10	0.03	0.10	0.05
	NormalizedGamma	saline	0.09	0.06	0.06	0.11	0.12	0.04	0.11	0.08
		PTZ	0.11	0.05	0.09	0.06	0.09	0.03	0.09	0.04
M1 cortex			Single				Multiple			
		Injections	Mean	SD	Median	IQR	Mean	SD	Median	IQR
	Occurrence	saline	0.49	0.32	0.45	0.23	0.55	0.39	0.48	0.46
		PTZ	0.71	0.39	0.68	0.30	0.48	0.45	0.34	0.59
	Duration	saline	1.27	0.48	1.07	0.53	1.45	0.62	1.32	0.82
		PTZ	1.51	0.39	1.43	0.45	1.56	0.53	1.42	0.92
	Amplitude	saline	-54.55	30.00	-44.00	38.00	-68.67	25.34	-78.50	48.25
		PTZ	-50.33	16.31	-47.00	18.50	-52.40	19.33	-50.00	32.00
	RectifiedArea	saline	0.17	0.14	0.12	0.13	0.10	0.05	0.12	0.10
		PTZ	0.14	0.05	0.13	0.08	0.11	0.06	0.11	0.10
	Up state Index	saline	0.08	0.09	0.06	0.06	0.06	0.06	0.06	0.08
		PTZ	0.10	0.06	0.09	0.08	0.05	0.05	0.04	0.08
	NormalizedDelta	saline	0.58	0.11	0.56	0.19	0.56	0.08	0.54	0.16
		PTZ	0.54	0.08	0.55	0.10	0.57	0.11	0.57	0.21
	NormalizedTheta	saline	0.14	0.05	0.13	0.05	0.18	0.05	0.17	0.10
		PTZ	0.15	0.03	0.15	0.05	0.15	0.02	0.15	0.03
	NormalizedAlpha	saline	0.06	0.02	0.06	0.02	0.06	0.02	0.06	0.03
		PTZ	0.07	0.02	0.06	0.04	0.06	0.03	0.06	0.04
	NormalizedBeta	saline	0.11	0.05	0.11	0.07	0.10	0.04	0.10	0.06
		PTZ	0.12	0.03	0.12	0.06	0.10	0.05	0.11	0.08
	NormalizedGamma	saline	0.12	0.07	0.14	0.15	0.09	0.06	0.08	0.11
		PTZ	0.13	0.04	0.14	0.05	0.12	0.06	0.11	0.08

Table 24. Data of all ten (10) parameters of spontaneous Up states per each experimental group presented as mean ± standard deviation (sd) and median with interquartile range (IQR). Although mean ± sd and median (IQR) are used to describe mainly normally and non-normally distributed data, respectively, we have included both descriptive for each parameter. The normality of distribution of data for each group can be seen in preceding table 23.

5.1.2. Three-way ANOVA

3-way ANOVA: number * cortex * injections

Between Subjects Factors

Factor	Level	N
Injections	Saline	39
	PTZ	35
Cortex	S1	43
	M1	31
Number Of Injections	single	39
	multiple	35

Source	Dependent variable	Sum of Squares	df	Mean Square	F	Sig.
Number Of Injections * Injections* Cortex	ARTOccurrenceNumberXCortexXInjections	6.182	1	6.182	.012	.912
	ARTDurationNumberXCortexXInjections	6.328	1	6.328	.013	.910
	ARTAmplitudeNumberXCortexXInjections	587.484	1	587.484	1.267	.264
	ARTRectifiedAreaNumberXCortexXInjections	6.467	1	6.467	.013	.908
	ART Up state IndexNumberXCortexXInjections	6.501	1	6.501	.015	.904
	NormalizedDelta	.004	1	.004	.449	.505
	NormalizedTheta	.015	1	.015	13.195	.001
	NormalizedAlpha	.001	1	.001	2.350	.130
	NormalizedBeta	.000	1	.000	.047	.829
	ARTNormalizedGammaNumberXCortexXInjections	1391.672	1	1391.672	2.864	.095
Error	ARTOccurrenceNumberXCortexXInjections	32987.442	66	499.810		
	ARTDurationNumberXCortexXInjections	32738.993	66	496.045		
	ARTAmplitudeNumberXCortexXInjections	30614.182	66	463.851		
	ARTRectifiedAreaNumberXCortexXInjections	31898.018	66	483.303		
	ART Up state IndexNumberXCortexXInjections	29307.996	66	444.061		
	NormalizedDelta	.649	66	.010		
	NormalizedTheta	.074	66	.001		
	NormalizedAlpha	.025	66	.000		
	NormalizedBeta	.111	66	.002		
	ARTNormalizedGammaNumberXCortexXInjections	32070.017	66	485.909		

Table 25. The significance of interaction between the effects of "number of injections", "type of cortex" and "injections" for all ten (10) parameters of spontaneous Up states. Data that were not normally distributed (Table 23) were previously transformed according to the Aligned Rank Transformation (ART) rules.

5.2. Simple effect analysis

5.2.1. Simple effects of seizures ("injections")

5.2.1.1. Simple effects of seizures on Theta

Estimated Marginal Means

Number Of Injections * Injections * Cortex

Estimates

Dependent Variable:NormalizedTheta

NumberOfInjections	Injections	Cortex	Mean	Std. Error	95% Confidence Interval	
					Lower Bound	Upper Bound
single	saline	S1	.167	.011	.145	.190
		M1	.136	.010	.116	.156
	PTZ	S1	.148	.011	.127	.169
		M1	.147	.011	.125	.169
multiple	saline	S1	.158	.009	.139	.176
		M1	.184	.014	.157	.211
	PTZ	S1	.208	.010	.187	.228
		M1	.146	.015	.116	.176

Pairwise Comparisons

Dependent Variable:NormalizedTheta

NumberOfInjections	Cortex	(I) Injections	(J) Injections	Mean Difference (I-J)	Std. Error	Sig.[a]	95% Confidence Interval for Difference[a]	
							Lower Bound	Upper Bound
single	S1	saline	PTZ	.020	.015	.205	-.011	.050
		PTZ	saline	-.020	.015	.205	-.050	.011
	M1	saline	PTZ	-.011	.015	.472	-.041	.019
		PTZ	saline	.011	.015	.472	-.019	.041
multiple	S1	saline	PTZ	-.050*	.014	.001	-.077	-.022
		PTZ	saline	.050*	.014	.001	.022	.077
	M1	saline	PTZ	.038	.020	.065	-.002	.078
		PTZ	saline	-.038	.020	.065	-.078	.002

Based on estimated marginal means

a. Adjustment for multiple comparisons: Bonferroni.

*. The mean difference is significant at the .05 level.

Univariate Tests

Dependent Variable:NormalizedTheta

NumberOfInjections	Cortex		Sum of Squares	df	Mean Square	F	Sig.
single	S1	Contrast	.002	1	.002	1.637	.205
		Error	.074	66	.001		
	M1	Contrast	.001	1	.001	.523	.472
		Error	.074	66	.001		
multiple	S1	Contrast	.015	1	.015	13.157	**.001**
		Error	.074	66	.001		
	M1	Contrast	.004	1	.004	3.525	.065
		Error	.074	66	.001		

Each F tests the simple effects of Injections within each level combination of the other effects shown. These tests are based on the linearly independent pairwise comparisons among the estimated marginal means.

5.3. *Analysis of two-way interaction: age of injections * injections*

5.3.1. *Normality of data distribution and descriptive of data for each group (age of injections * injections)*

Number of Injections		Single			Multiple		
		Shapiro-Wilk			Shapiro-Wilk		
	Injections	Statistic	df	Sig.	Statistic	df	Sig.
Occurrence	saline	.833	20	.003	.893	19	.037
	PTZ	.916	19	.097	.924	16	.197
Duration	saline	.926	20	.132	.917	19	.102
	PTZ	.929	19	.165	.947	16	.441
Amplitude	saline	.572	20	.000	.917	19	.100
	PTZ	.810	19	.002	.921	16	.173
RectifiedArea	saline	.800	20	.001	.790	19	.001
	PTZ	.969	19	.752	.781	16	.002
NetworkIndex	saline	.708	20	.000	.457	19	.000
	PTZ	.783	19	.001	.656	16	.000
NormalizedDelta	saline	.988	20	.995	.959	19	.556
	PTZ	.967	19	.719	.956	16	.593
NormalizedAlpha	saline	.900	20	.040	.923	19	.128
	PTZ	.921	19	.119	.973	16	.883
NormalizedBeta	saline	.958	20	.509	.948	19	.361
	PTZ	.974	19	.850	.982	16	.976
NormalizedGamma	saline	.915	20	.080	.955	19	.487
	PTZ	.940	19	.266	.859	16	.019

Table 26. Output (significance) of Shapiro-Wilki's normality tests of distribution of data within each group. Data is color-coded darker colors indicating lower p values.

Number of Injections		Single				Multiple			
	Injections	Mean	SD	Median	IQR	Mean	SD	Median	IQR
Occurrence	saline	0.88	0.74	0.52	0.96	1.56	0.43	1.45	0.40
	PTZ	1.10	0.71	0.83	0.99	1.39	0.41	1.33	0.62
Duration	saline	1.38	0.44	1.37	0.64	-63.05	26.61	-60.00	36.00
	PTZ	1.49	0.34	1.52	0.38	-67.00	29.64	-61.00	38.00
Amplitude	saline	-69.10	62.28	-49.00	37.50	0.13	0.07	0.12	0.06
	PTZ	-59.68	25.58	-50.00	20.00	0.13	0.11	0.11	0.09
RectifiedArea	saline	0.19	0.13	0.16	0.09	0.17	0.26	0.10	0.10
	PTZ	0.16	0.06	0.16	0.09	0.14	0.18	0.08	0.11
NetworkIndex	saline	0.19	0.24	0.08	0.20	0.54	0.08	0.52	0.12
	PTZ	0.21	0.20	0.14	0.16	0.54	0.09	0.55	0.14
Normalized Delta	saline	0.58	0.12	0.57	0.16	0.06	0.01	0.06	0.02
	PTZ	0.56	0.10	0.58	0.13	0.07	0.02	0.07	0.03
Normalized Alpha	saline	0.06	0.02	0.07	0.02	0.12	0.04	0.12	0.07
	PTZ	0.06	0.02	0.05	0.03	0.10	0.03	0.10	0.05
Normalized Beta	saline	0.11	0.05	0.11	0.07	0.11	0.05	0.10	0.08
	PTZ	0.11	0.04	0.10	0.05	0.10	0.04	0.09	0.03
Normalized Gamma	saline	0.11	0.07	0.08	0.12	0.00	0.00	0.00	0.00
	PTZ	0.12	0.04	0.11	0.05	0.00	0.00	0.00	0.00

Table 27. Up states parameters per each experimental group presented as mean ± standard deviation (SD) and median with interquartile range (IQR). Although mean ± sd and median (IQR) are used to describe mainly normally and non-normally distributed data, respectively, we have included both descriptive for each parameter. The normality of distribution of data for each group can be seen in preceding table 26.

5.3.2. *Two-way ANOVA*

2-way ANOVA: number of injections * injections

Between Subjects Factors

Factor	Level	N
Injections	Saline	39
	PTZ	35
Number Of Injections	single	39
	multiple	35

Source	Dependent variable	Sum of Squares	df	Mean Square	F	Sig.
Number Of Injections * Injections	ARTOccurrenceNumberXInjections	1189.941	1	1189.941	2.564	.114
	Duration	.388	1	.388	2.349	.130
	ARTAmplitudeAgeXInjections	5.457	1	5.457	.012	.914
	ARTRectifiedAreaNumberXInjections	49.155	1	49.155	.102	.750
	ARTNetworkIndexNumberXInjections	1096.810	1	1096.810	2.386	.127
	NormalizedDelta	.001	1	.001	.150	.700
	ARTNormalizedAlphaNumberXInjections	295.931	1	295.931	.623	.432
	NormalizedBeta	.002	1	.002	1.474	.229
	ARTNormalizedGammaNumberXInjections	771.680	1	771.680	1.639	.205
Error	ARTOccurrenceNumberXInjections	32483.392	70	464.048		
	Duration	11.575	70	.165		
	ARTAmplitudeAgeXInjections	32890.540	70	469.865		
	ARTRectifiedAreaNumberXInjections	33665.909	70	480.942		
	ARTNetworkIndexNumberXInjections	32180.980	70	459.728		
	NormalizedDelta	.670	70	.010		
	ARTNormalizedAlphaNumberXInjections	33234.747	70	474.782		
	NormalizedBeta	.116	70	.002		
	ARTNormalizedGammaNumberXInjections	32953.266	70	470.761		

Table 28. The significance of interaction between the effects of "number of injections" and "injections" for nine parameters of spontaneous Up states. Data that were not normally distributed (Table 26) were previously transformed according to the Aligned Rank Transformation (ART) rules.

6. REFERENCES

Wobbrock JO, Findlater L, Gergle D, Higgins J. 2011. The aligned rank transform for non parametric factorial analyses using only ANOVA procedures. In. Proceedings of the ACM Conference on Human Factors in Computing Systems (CHI'11) Vancouver, BC; New York, NY: ACMPress p 143–146.
