# Supplementary material for: Long-Term Effects of Early Life Seizures on Endogenous Local Network Activity of the Mouse Neocortex
Source: Front Synaptic Neurosci. 2018 Nov 27;10:43. doi: 10.3389/fnsyn.2018.00043 (PMC6277496; doi:10.3389/fnsyn.2018.00043)
Supplement: Supplementary file 2 [file Data_Sheet_2.PDF]

SUPPLEMENTARY FIGURE 1

Quantification of the local field potential (LFP) Up states.(A) Continuous LFP recording (1–200Hz) of spontaneous Up states from a cortical slice. **(B)** *Top panel*: Automatically detected Up states of the signal in **(A)** are outlined by gray rectangles. *Bottom panel*: Individual up state at higher time resolution. Gray line is the automatically detected onset and offset of the event, on the basis of which duration is calculated. **(C)** Rectified signal (absolute valued signal) of the Up state, from which the rectified area is calculated. Modified from Rigas et al. (2015) Fig.1.
