# Supplementary figures and images for: Long-Term Effects of Early Life Seizures on Endogenous Local Network Activity of the Mouse Neocortex
Source: Front Synaptic Neurosci. 2018 Nov 27;10:43. doi: 10.3389/fnsyn.2018.00043 (PMC6277496; doi:10.3389/fnsyn.2018.00043)

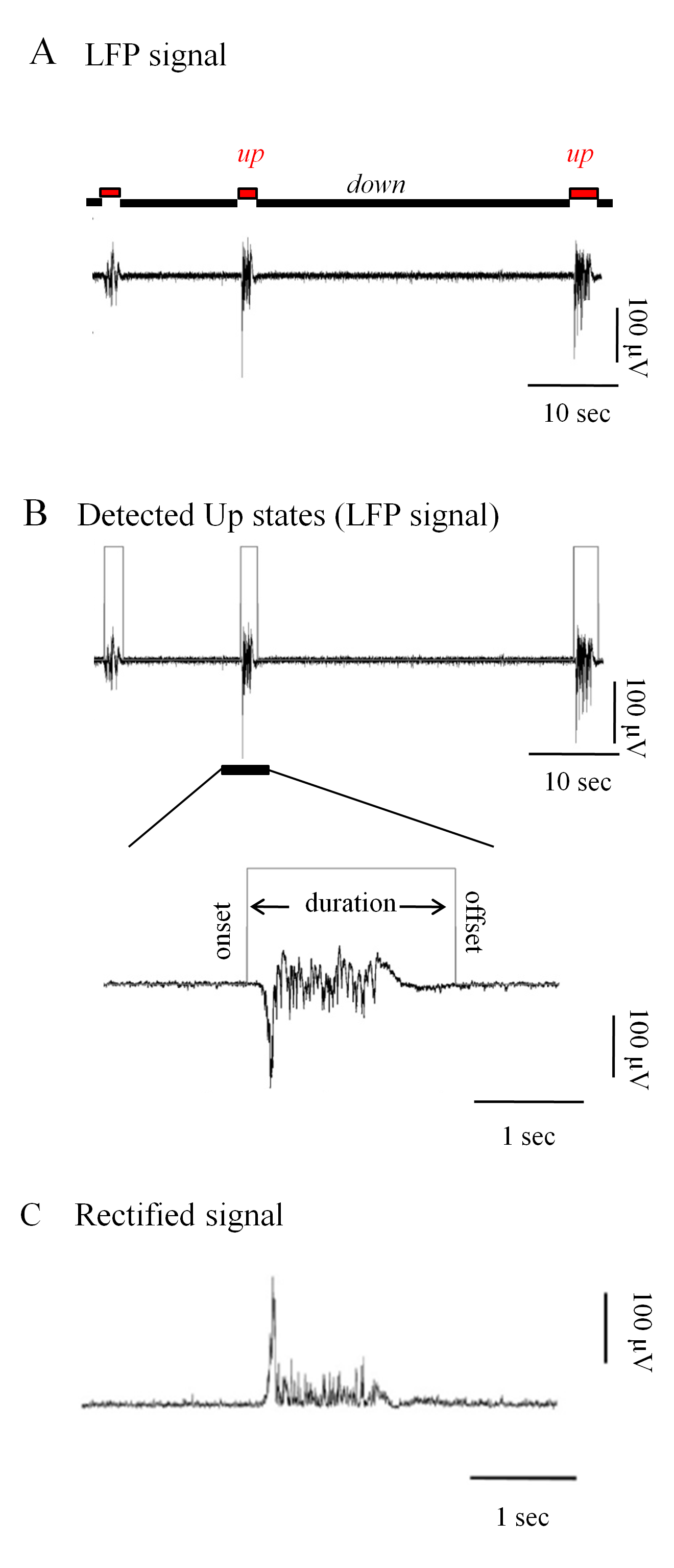

Supplement: Supplementary file 3 [file Image_1.TIF]
